# Efficient low-temperature wastewater treatment by *Pseudomonas zhanjiangensis* sp. nov.: a novel cold-tolerant bacterium isolated from mangrove sediment

**DOI:** 10.3389/fmicb.2024.1491174

**Published:** 2024-10-31

**Authors:** Ming Li, Xixi Hu, Tiancheng Ni, Yuan Ni, Changran Li, Dong Xue, Feng Li

**Affiliations:** ^1^School of Integrated Chinese and Western Medicine, School of Life Sciences, Anhui University of Chinese Medicine, Hefei, China; ^2^School of Life Sciences, Nanjing University, Nanjing, China

**Keywords:** wastewater treatment, cold tolerant, nitrogen removal, *Pseudomonas zhanjiangensis*, comparative genomic analysis

## Abstract

A novel heterotrophic, cold-tolerant bacterium, designated *Pseudomonas zhanjiangensis* 25A3E^T^, was isolated from mangrove sediment and demonstrated excellent efficiency in cold wastewater treatment. Phylogenetic analysis based on 16S rRNA gene sequences positioned strain 25A3E^T^ within the genus *Pseudomonas*, showing the highest similarity (98.7%) with *Pseudomonas kurunegalensis* LMG 32023^T^. Digital DNA–DNA hybridization (dDDH) and average nucleotide identity (ANI) values were below the species delineation thresholds (70% for dDDH, 95% for ANI), indicating that strain 25A3E^T^ represents a novel species. This strain demonstrated high efficiency in removing nitrogen (N) and organic pollutants under low-temperature conditions. Specifically, it achieved 72.9% removal of chemical oxygen demand (COD), 70.6% removal of ammoniacal nitrogen (NH_4_^+^-N), and 69.1% removal of total nitrogen (TN) after 96 h at 10°C. Genomic analysis identified key genes associated with cold adaptation, nitrogen removal and organic matter degradation. These findings indicate that *Pseudomonas zhanjiangensis* 25A3E^T^ holds significant potential for application in cold temperature wastewater treatment, offering a promising solution for environmental remediation in regions with low ambient temperatures.

## 1 Introduction

Water eutrophication has become an increasing severe issue in recent years due to the excessive discharge of organic matter and nitrogen, which destabilizes ecosystem (Lefebvre and Moletta, 2006; Basu et al., 2022). Therefore, effective wastewater treatment is essential before discharging it into natural water bodies. Among the various treatment method, biological wastewater treatment is widely favored for its simplicity, low cost, mild operational conditions, and minimal secondary pollution (Khin and Annachhatre, 2004; Wu et al., 2020).

Mangroves are intertidal wetlands that play crucial ecological roles in tropical and subtropical coastlines worldwide (Duke et al., 2007; Sheaves, 2009), including organic pollutant degradation, nutrient cycling, pollution trapping, and surface runoff storage (Wei-dong, 2003; Hou et al., 2024). Previous studies have demonstrated that mangroves are uniquely rich in microbial diversity, which significantly contributes to their ecosystem dynamics (Thatoi et al., 2013). Various bacterial genera and functional microorganisms, such as sulfate reducers (Zhou et al., 2023), nitrate reducers (Ye et al., 2020), denitrifiers (Fan et al., 2024), and hydrocarbon degraders (Marasco et al., 2023), have been reported from mangrove sediment. At present, some new species have been isolated from mangrove sediments with pollutant-degrading capabilities (Mukherji et al., 2022; Hu et al., 2023; Marasco et al., 2023). Yet, few studies have focused on bacteria isolated from subtropical mangroves for low-temperature wastewater treatment. Strains employed for low-temperature wastewater treatment are generally isolated from cold environments (Cavicchioli et al., 2002; Xu et al., 2018; Huang et al., 2022).

*Pseudomonas* is a genus of Gram-*negative* bacteria with species that have been isolated from diverse environments, including soil, plants, animals, and water (Nicklasson et al., 2022; Carlier et al., 2024; Ge et al., 2024; Lick et al., 2024). Members of this genus exhibit remarkable metabolic and functional diversity (Gross and Loper, 2009), enabling them to thrive in a wide range of habitats (Silby et al., 2011). Several strains with this genus, particularly those isolated from contaminated environments such as polluted soil and wastewater, have demonstrated potential in pollutant degradation. Notable examples include *Pseudomonas stutzeri* (Huang et al., 2015), *Pseudomonas chengduensis* (Peng et al., 2023; Yi et al., 2023), *Pseudomonas hibiscicola* (An et al., 2021), *Pseudomonas indoloxydans* (Manickam et al., 2008; Shahid et al., 2020), *Pseudomonas indoloxydans* (Guo et al., 2018), *Pseudomonas mendocina* (Zhang et al., 2023b), and *Pseudomonas glycinae* (Tong et al., 2023), all of which have been applied in wastewater treatment processes. However, most strains typically exhibit optimal nitrogen and organic matter removal at room temperature, with their metabolic activities significantly diminished at lower temperatures (Li et al., 2015; Lei et al., 2016; Lin et al., 2020). This limitation leads to reduced pollutant removal efficiency under cold conditions. To address this challenge, the use of cold-tolerant microorganisms capable of maintaining high metabolic activity across a broad temperature range is of considerable importance for enhancing the efficacy of biological wastewater treatment.

In this study, we isolated and characterized a novel cold-tolerant strain, designated 25A3E^T^, from the mangrove sediment. The strain underwent polyphasic taxonomic characterization and its potential for removing chemical oxygen demand (COD), ammonium nitrogen (NH_4_^+^-N), and total nitrogen (TN) at 10°C was assessed. Genome analysis was conducted to identify genes associated with low-temperature wastewater degradation, and comparative genomic analysis was employed to further elucidate the ecological role of strain 25A3E^T^ in comparison to other related type strains. The insights gained from this study could contribute to the development of more efficient wastewater treatment strategies, particularly in cold environments, thereby improving environmental sustainability.

## 2 Materials and methods

### 2.1 Sampling, isolation, and cultivation

A sediment sample was collected from the mangrove ecosystem in Zhanjiang, Guangdong Province, China (109°90′ N, 20°40′ E). 10 g of the sediment were added to 100 mL 0.9% NaCl (w/v) sterile solution and thoroughly mixed by vigorous shaking for 30 min. The resulting suspension was serially diluted, and an aliquot (100 μL) of each dilution was spread onto 2216E agar plates (5 g Peptone, 5 g NaCl, 1 g yeast, 15 g agar, 1,000 mL sterile seawater, pH 7.8). The plates were incubated at 30°C for 5 days. Single colonies were isolated and repeatedly streaked onto fresh 2216E agar plates under aerobic conditions at 30°C. Pure colonies were obtained after 1 week of incubation and were stored in 2216E broth supplemented with 50% (v/v) glycerol at −80°C.

### 2.2 Molecular identification

Genomic DNA from strain 25A3E^T^ was extracted with a commercial bacterial genomic DNA kit (TIANGEN, China) according to the manufacturer’s instructions. The 16S rRNA gene sequence was amplified using the universal bacterial primers 27F and 1492R (Kitahara et al., 2012). The obtained 16S rRNA gene sequence was submitted to EzBioCloud^1^ (Yoon et al., 2017) for species identification by comparing it to closely related sequences. Multiple sequence alignments were performed using Clustal W (Larkin et al., 2007). Phylogenetic analyses based on 16S rRNA gene sequences were conducted using the software MEGA X (Kumar et al., 2018). Phylogenetic trees were constructed using the neighbor-joining (NJ) (Naruya Saitou, 1987), minimum-evolution (ME) (Pardi et al., 2010), and maximum likelihood (ML) (Felsenstein, 1981) methods, applying the Kimura two-parameter model (Kimura, 1980) with 1,000 bootstrap replications. *Azomonas agilis* NCIB 11693^T^ was used as an outgroup.

### 2.3 Phenotypic and chemotaxonomic characterization

The cell morphology of strain 25A3E^T^ was observed using transmission electron microscopy (TEM) (Hitachi HT7700, Japan). Catalase and oxidase activities were assessed using 3% (v/v) hydrogen peroxide and 1% (w/v) tetramethyl-p-phenylenediamine, respectively. Gram staining was performed using a Gram staining kit (Solarbio Life Science, China). The strains growth was tested at various temperatures ranging from 0°C to 45°C (0, 4, 10, 15, 20, 25, 30, 35, 37, 40 and 45°C) over 1 week. NaCl tolerance was evaluated on 2216E agar containing NaCl concentrations from 0 to 10% (w/v) in 0.5% increments. The pH range for growth was determined using media with pH 4.0 to 11.0 in 0.5-unit intervals, following the buffer system described by Xie et al. (2024). The hydrolysis of cellulose, Tweens (20, 40, and 80), starch, and casein was evaluated as previously described (Tindall et al., 2014). Anaerobic growth was assessed in 2216E medium at 30°C for 7 days using the Anaero Pack gas system (Anaero Pack disposable, Mitsubishi Gas Chemical, Tokyo, Japan). Enzymatic activities and other physiological and biochemical traits were tested using API ZYM and API 20NE stripes (bioMérieux, France) according to the manufacturer’s instructions. Carbon source assimilation of strain 25A3E^T^ were determined GEN III MicroPlates (Biolog, USA).

For chemotaxonomic analysis, strain 25A3E^T^ was grown on 2216E agar for 3 days at 30°C until reaching the post-growth stage. Polar lipids were extracted following the method described by Minnikin et al. (1979) and analyzed by two-dimensional thin-layer chromatography (Collins and Jones, 1980). Cellular fatty acids were extracted and analyzed according to the standard protocol of the Microbial Identification System (MIDI) using a gas chromatograph system (model 6,890, Agilent, USA) (Fykse et al., 2015).

### 2.4 Evaluation of organic matter and nitrogen removal efficiency

Synthetic wastewater was prepared to mimic the composition of natural wastewater, containing (g/L): glucose, 0.34; soluble starch, 0.32; tryptone, 0.316; beef extract 0.12; KH_2_PO_4_, 0.07; (NH_4_)_2_SO_4_, 0.0284; NH_4_Cl, 0.45; CH_3_COONa, 0.466; KNO_3_, 0.1; and, Na_2_CO_3_, 0.06. The pH of the synthetic wastewater was adjusted to 7.6. The concentrations of COD, NH_4_^+^-N, and TN in the synthetic wastewater were approximately 1,200 mg/L, 120 mg/L, and 140 mg/L, respectively (Wu et al., 2020). Strain 25A3E^T^ was inoculated into 2216E medium and cultured for 24 h. Following centrifugation, the bacterial suspensions were adjusted to an OD_600_ of 1.0 using 0.9% sodium chloride solution. A 10% inoculum was introduced into synthetic wastewater, with an initial OD_600_ value of approximately 0.05. The cultures were then incubated on a shaker at 10°C and 150 rpm. Samples were collected at various time points, and the concentrations of COD, NH_4_^+^-N, and TN were measured using standard methods (Ai et al., 2019). The optical density (OD_600_) of the cells was monitored by measuring the absorbance at 600 nm with a spectrophotometer (UNICO, UV-2365, China). All experiments were performed in triplicate, and average values were calculated.

### 2.5 Comparative genomic analyses

The genomes of strain 25A3E^T^ was sequenced using the Illumina Hiseq 4,000 platform by Sangon Biotech (Shanghai, China). Sequence assembly was performed using SPAdes version 3.5.0 (Bankevich et al., 2012). Additional genome sequences used in this study were retrieved from the GenBank database. Gene prediction was conducted with Prokka (v1.13.7), and functional annotation was achieved using Kyoto Encyclopedia of Genes and Genomes (KEGG) databases (Kanehisa and Goto, 2000). Genome completeness and contamination were assessed with CheckM v1.2.2 (Parks et al., 2015). Visual genome homology comparisons were performed using with BRIG (BLAST Ring Image Generator)^2^ with default settings (Alikhan et al., 2011). Orthologous genes were identified with the USEARCH algorithm using a threshold of 0.5. The Ortho Average Nucleotide Identity (OrthoANI) between strain 25A3E^T^ and type strains was calculated using the OrthoANI Tool (OAT) (Lee et al., 2016), while digital DNA–DNA hybridization (dDDH) values were determined using the Genome-to-Genome Distance Calculator^3^ (Meier-Kolthoff et al., 2013).

To construct a robust phylogeny, a well-characterized core gene set, bac120 (comprising 120 genes within the domain Bacteria) (Parks et al., 2017), was employed to generate a genome-based ML phylogenetic tree. Genome sequence data of strain 25A3E^T^, along with related *Pseudomonas* species, were processed using the Easy-CGTree version 4.0 Perl script^4^ to clarify the phylogenetic relationships (Zhang et al., 2023a). This approach facilitated the construction of a phylogenomic tree, providing insights into the evolutionary connections and taxonomic positioning of strain 25A3E^T^ relative to closely related species.

To assess the functional capacities and metabolic activities of these microbial species, the METABOLIC (Metabolic and Biogeochemistry Analyses in Microbes)^5^ tool was employed (Zhou et al., 2022). Specifically, the METABOLIC-G module was used to analyze individual genome sequences. The workflow began with protein-coding genes prediction using Prodigal (Hyatt et al., 2010), followed by comparison against HMM-based databases using the hmmsearch tool from the HMMER package (Hyatt et al., 2010). The databases included KOfam (Aramaki et al., 2020), TIGRfam (Selengut et al., 2007), Pfam (Finn et al., 2008), and custom metabolic HMM profiles (Anantharaman et al., 2016). A subset of protein families was validated through motif-checking to ensure accuracy. The findings were compiled into an Excel spreadsheet, detailing the presence or absence of key metabolic marker proteins, functional traits, KEGG module steps, and hits for carbohydrate-active enzymes (CAZymes) and peptidases/inhibitors.

### 2.6 Identification of key nitrogen removal and cold stress adaptation enzyme genes based on genome analysis

Key enzymes involved in nitrogen removal and cold stress adaptation were identified through genome annotation and subsequent analysis using Protein BLAST (BLASTp) in the NCBI database and PowerBlast software. These analyses focused on functional enzymes related to the removal of COD, NH_4_^+^-N, TN, and cold adaptation. Relevant functional genes were identified by scanning the genome of strain 25A3E^T^.

## 3 Results and discussion

### 3.1 Isolation, selection and identification of strains

In this study, 52 isolates were obtained from mangrove sediment, among which strain 25A3E^T^ exhibited significant COD, NH_4_^+^-N, and TN removal activities. Additionally, strain 25A3E^T^ demonstrated the ability to grow at low temperatures while maintaining its effectiveness in removing these pollutants.

**Figure 1:**
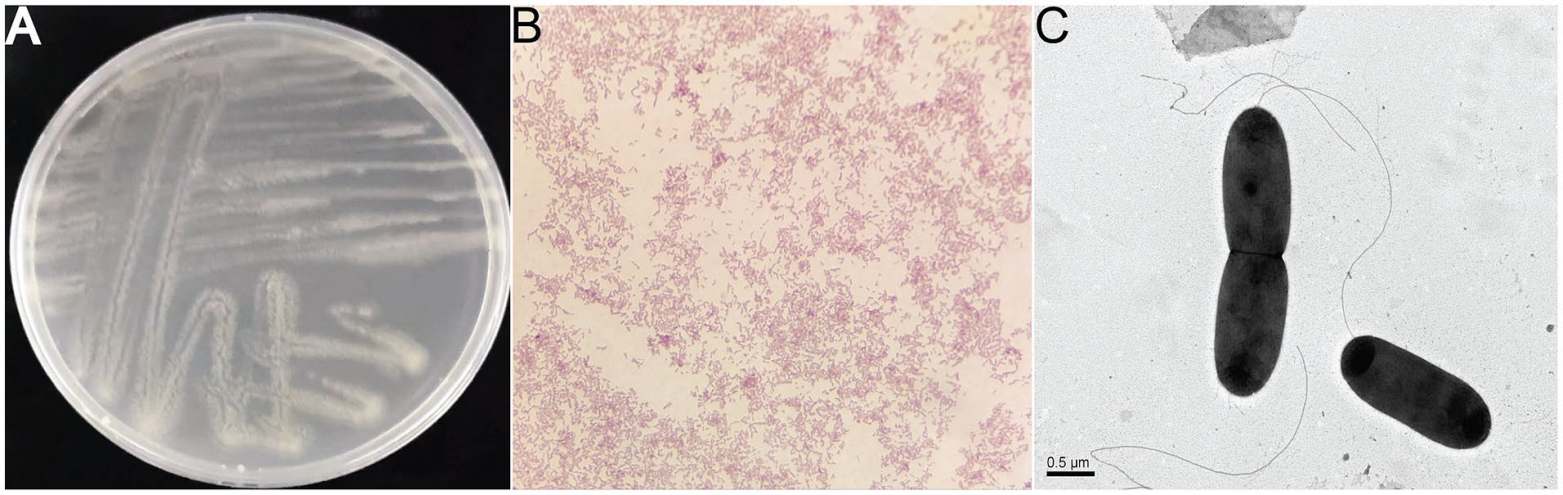
The morphologies of the strain 25A3E^T^. **(A)** Colony on 2216E plates; **(B)** microscopic observation of Gram staining; **(C)** transmission electron micrograph showing the cell morphology of strain 25A3E^T^, Bar, 0.5 μm.

On 2216E plate incubated at 30°C, colonies of strain 25A3E^T^ appeared irregular, diffuse, and translucent. The cells of strain 25A3E^T^ were Gram-negative, facultatively anaerobic, motile, and rod-shaped, measuring 0.75 μm in width and 1.5–1.75 μm in length (Figure 1). Growth was observed at temperatures ranging from 4 to 37°C (optimal at 25°C), at pH values from 6 to 10 (optimal pH 8), and in NaCl concentrations from 0 to 8% (optimal at 0.5%) (Supplementary Figure S1).

**Figure 2:**
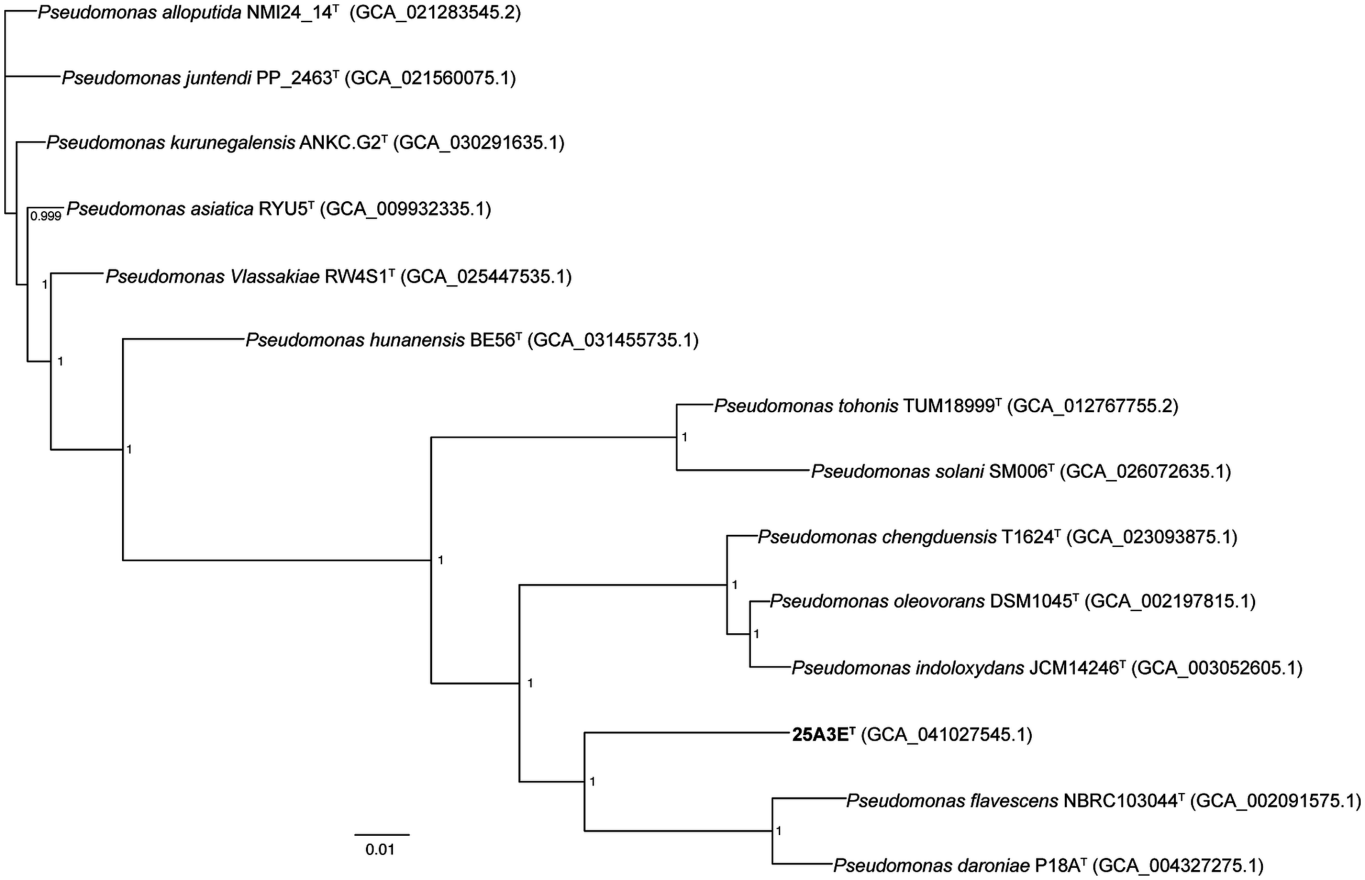
Maximum-likelihood phylogenetic tree based on the bac120 gene set showing the phylogenetic relationship of 25A3E^T^ in the genus *Pseudomonas*. Bootstrap values based on 1,000 replicates were shown at the branch points nodes. The RefSeq assembly accession number is indicated in the bracket. Bar, 0.01 substitutions per nucleotide position.

The 16S rRNA gene sequence of strain 25A3E^T^ consisting of 1,466 base pairs, was determined in this study. The closest match for the 16S rRNA gene sequence was *Pseudomonas kurunegalensis* LMG 32023 ^T^ with a similarity of 98.7%, which is at the species delineation threshold of 98.7% (Chun et al., 2018). A comparative genomic analysis, including the construction of a phylogenetic tree based on 120 core genes, was conducted to further clarify the taxonomic position of strain 25A3E^T^ (Figure 2). In the phylogenetic tree based on the NJ algorithm, strain 25A3E^T^ formed a distinct lineage within the genus *Pseudomonas* (Supplementary Figure S2). This distinct phylogenetic positioning was also observed in trees constructed using the ME and ML methods (Supplementary Figures S3, S4). The phylogenetic tree based on 120 conserved genes and 16S rRNA genes suggested that strain 25A3E^T^ is closely related to *P. chengduensis* T1624^T^ (Tao et al., 2014), *P. oleovorans* DSM 1045^T^ (Saha et al., 2010), *P. tohonis* TUM18999^T^ (Yamada et al., 2021), *P. daroniae* P18A^T^ (Bueno-Gonzalez et al., 2019), *P. flavescens* NBRC103044^T^ (Hildebrand et al., 1994), *P. solani* Sm006^T^ (Sawada et al., 2023), and *P. indoloxydans* JCM 14246^T^ (Manickam et al., 2008). These strains formed a tight cluster, and they were selected as reference species for further comparative analysis.

The ANI values and dDDH values between strain 25A3E^T^ and the reference strains ranged from 79.7 to 80.6% and from 23.4 to 23.9%, respectively (Supplementary Figure S5). These values were significantly lower than the species delineation threshold, which were 95–96% for ANI and 70% for dDDH (Goris et al., 2007; Meier-Kolthoff et al., 2013; Chun et al., 2018). These results confirmed that 25A3E^T^ represents a novel species within the genus *Pseudomonas*.

### 3.2 Physiology and chemotaxonomic characterization

Strain 25A3E^T^ was found to be positive for both catalase and oxidase. It demonstrated the ability to hydrolyze starch and Tween 80, but not gelatin, cellulose and Tween 20. According to the API 20NE test, the strain showed positive results for nitrate reduction to nitrites and esculin hydrolysis, and it was able to assimilate glucose, maltose, gluconate, caprate, malate, and citrate. In the API ZYM test, strain 25A3E^T^ tested positive for alkaline phosphatase, esterase (C4), esterase lipase (C8), valine arylamidase, and acid phospha-tase. The oxidations of the sole carbon source (Biolog) were positive for D-maltose, sucrose, *α*-D-glucose, fusidic acid, glycerol, D-serine, L-alanine, L-arginine, L-aspartic acid, L-glutamic acid, L-histidine, L-serine, D-gluconic acid, L-lactic acid, citric acid, α-keto-glutaric acid, D-malic acid, L-malic acid, nalidixic acid, lithium chloride, potassium tellurite, Tween 40, *γ*-amino-butryric acid, *β*-hydroxy-D, L-butyric acid, propionic acid, acetic acid, and formic acid. The differential physiological and bio-chemical characteristics of strain 25A3E^T^ and reference strains were provided in Table 1.

**Table 1:** Differential characteristics of strain 25A3E^T^ and most closely related species.

Characteristic	1	2	3	4	5	6	7	8
Cell size (μm)	0.75 × 1.5–1.75	1.1–1.8 × 0.4–0.5	0.8–1.0 × 2.0–2.5	NA	2.2 ± 0.4 × 1.0 ± 0.08	1.0–2.0 × 0.5–0.8	1.7 × 0.4	0.6–0.7 × 1.6–2.3
Growth Type	Facultatively anaerobic	Facultatively anaerobic	Aerobic	NA	Aerobic	Aerobic	Aerobic	Aerobic
Motility	motile	non-motile	motile	NA	motile	motile	motile	motile
Temperature range (°C)	4–37	4–42	10–42	NA	12–37	20–42	4–37	*NA*
NaCl range (%, w/v)	0–8	0–8	2.5–5.0	NA	0–5	1–5	NA	*NA*
pH range	6–10	6–10	6.5–9.0	NA	6–8	5.5–9.5	6–9	*NA*
Starch hydrolysis	+	−	−	−	NA	NA	NA	−
Gelatin hydrolysis	−	+	+	−	+	+	−	−
Nitrate reduction	+	+	+	+	−	+	−	NA
Tweens 80	+	+	+	−	NA	NA	NA	−
Sugar utilization								
D-Trehalose	−	−	NA	−	−	−	−	+
Sucrose	+	−	NA	−	−	−	−	+
D-Salicin	−	+	NA	−	−	NA	NA	−
α-D-Glucose	+	+	−	NA	NA	+	+	NA
D-Mannose	−	−	NA	−	−	−	+	+
D-Mannitol	−	−	−	−	−	−	+	+
L-Pyroglutamic Acid	−	+	+	−	−	NA	+	NA
D-Gluconic Acid	+	+	−	−	−	NA	+	NA
Quinic Acid	−	−	−	+	+	NA	+	NA
D-Saccharic Acid	−	+	−	−	−	NA	+	NA

Strains: 1, Strain 25A3E^T^; 2, *P. chengduensis* T1624^T^ (Tao et al., 2014); 3, *P. indoloxydans* JCM 14246^T^ (Manickam et al., 2008); 4, P. oleovorans DSM 1045^T^ (Saha et al., 2010); 5, *P. solani* Sm006^T^ (Sawada et al., 2023); 6, *P. tohonis* TUM18999^T^ (Yamada et al., 2021); 7, *P. daroniae* P18A^T^ (Bueno-Gonzalez et al., 2019); 8, *P. flavescens* NBRC103044^T^ (Hildebrand et al., 1994). Symbol: +, positive; −, negative; w, weakly positive; NA, no data available.

Polar lipids of strain 25A3E^T^ were composed of phosphatidylglycerol (PG), diphosphatidylglycerol (DPG), phosphatidylethanolamine (PE), phosphatidylcholine (PC), sphingoglycolipid (SGL), phospholipids (PL), and unidentified lipids (Supplementary Figure S6). The major cellular fatty acid (>10%) were C_16:0_ (25.6%), C_17:0_ cyclo (12.2%), Summed feature 3 (16.7%), and Summed feature 8 (19.6%), the fatty acid profile was similar to those of the closest phylogenetic relatives, however, certain components exhibit differences. The major fatty acid profiles were largely consistent with those of related type strains within the genus *Pseudomonas*. Detailed fatty acid profiles were presented in Supplementary Table S1.

### 3.3 Evaluation of organic matter and nitrogen removal efficiency

**Figure 3:**
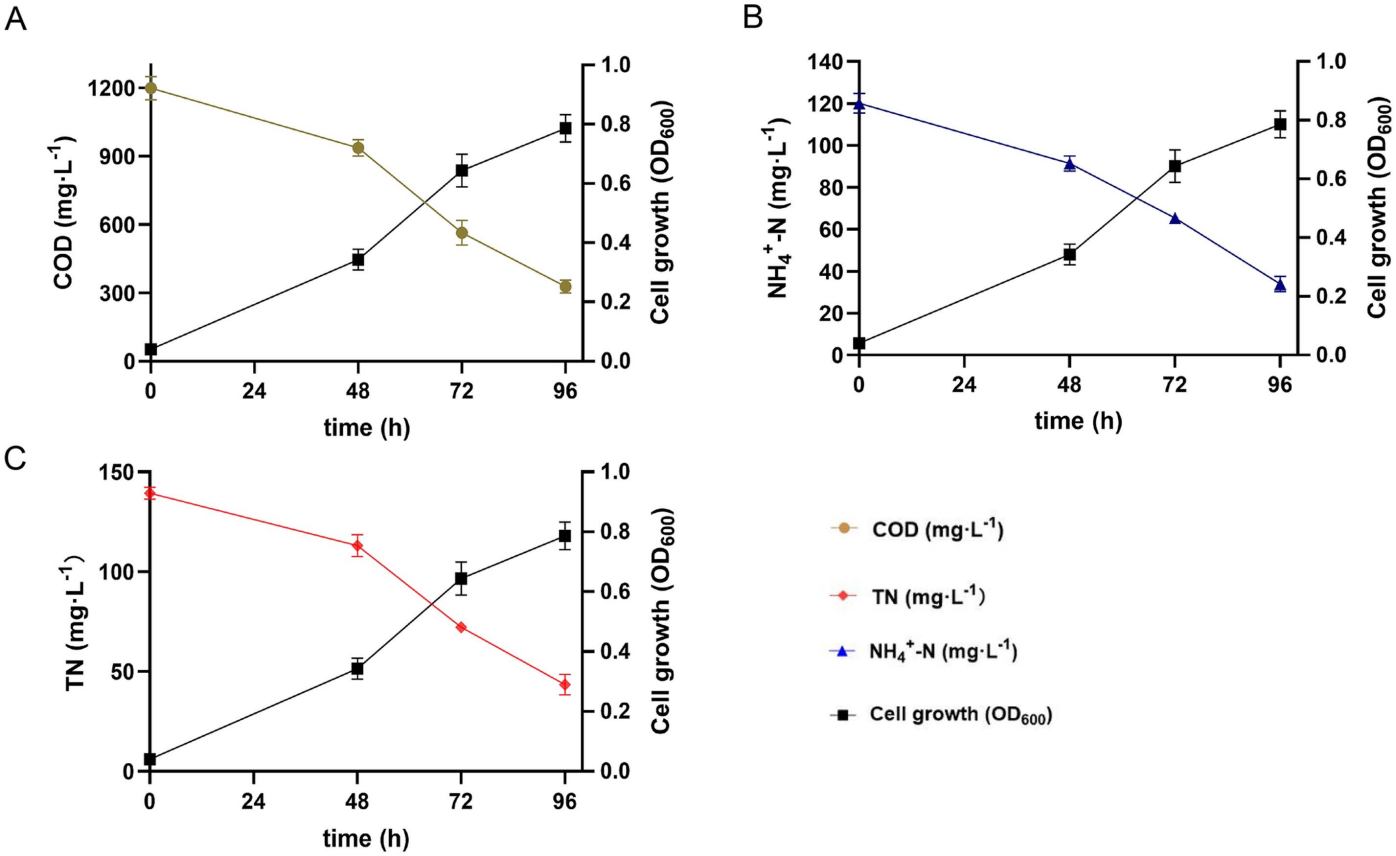
COD, NH_4_^+^-N, and TN removal characteristics of strain 25A3E^T^ in the artificial wastewater culture medium at 10°C, respectively. Values are means ± SE (standard error) for three replicates. **(A)** COD, chemical oxygen demand; **(B)** NH_4_^+^-N, Ammonium nitrogen; **(C)** TN, total nitrogen.

The COD, NH_4_^+^-N, and TN removal efficiencies of strain 25A3E^T^ over different time intervals (0, 48, 72, and 96 h) in an artificial wastewater culture medium at 10°C were shown in Figure 3. As shown in Figure 3A, the COD concentration decreased from 1,200 mg/L to 325 mg/L within 96 h, achieving a removal efficiency of 72.9%. Similarly, Figure 3B illustrates that the NH_4_^+^-N concentration dropped from 120 mg/L to 35.2 mg/L over the same period, corresponding to a removal efficiency of 70.6%. Figure 3C showed that the TN concentration decreased from 140 mg/L to 43.2 mg/L within 96 h, resulting in a removal efficiency of 69.1%. These results suggested that strain 25A3E^T^ has significant potential for the treatment of a wide range of pollutants in wastewater, particularly in cold environments. Among the pollutants tested, strain 25A3E^T^ demonstrated the highest removal efficiency for COD, followed by NH_4_^+^-N and TN. It is noteworthy that strains capable of simultaneously removing COD, NH_4_^+^-N, and TN at low temperatures were rarely reported, highlighting the unique capabilities of strain 25A3E^T^.

### 3.4 Genomic characterization and analysis

To further investigate the wastewater treatment capability of the cold-tolerant strain 25A3E^T^, a comprehensive genomic analysis was conducted. The genome of strain 25A3E^T^ consists of a single circular chromosome of 4,696,747 bp with a G + C content of 65.5 mol%. The genome contains 4,272 protein-coding genes, 58 tRNAs, and 7 rRNA genes. The genomic characteristics of 25A3E^T^ were compared with those reference strains in Supplementary Table S2.

Annotations from the KEGG database were summarized in Supplementary Figure S7. A total of 3,881 genes were annotated, representing 48% of the genome. The largest category of annotated genes was related to metabolism, comprising 2,373 genes (61.1% of the total annotated genes). These included genes involved in carbohydrate metabolism (467), energy metabolism (243), lipid metabolism (206), nucleotide metabolism (151), amino acid metabolism (498), as well as general metabolic overviews (485), and the metabolism of cofactors and vitamins (217). The genes associated with nucleotide metabolism (151) and amino acid metabolism (498) may play roles in nitrogen metabolism in strain 25A3E^T^. The large number of genes involved in carbohydrate metabolism indicates a strong capacity for carbohydrate utilization, which is consistent with the carbon source utilization observed in the Biolog GEN III MicroPlates test. This metabolic capacity is likely associated with the strain’s ability to efficiently degrade organic matter. These metabolic pathways suggest that strain 25A3E^T^ has considerable potential for environmental remediation.

### 3.5 Comparative genomic analyses with related species

The completeness and contamination of the genomes of the seven closely related strains were assessed, with values ranging from 99.16 to 100% and 0.48 to 2.47%, respectively, indicating the high quality of these genomes. A circular map comparing the genome of strain 25A3E^T^ to related species is shown in Supplementary Figure S8, indicating that they share many conserved genomic regions.

**Figure 4:**
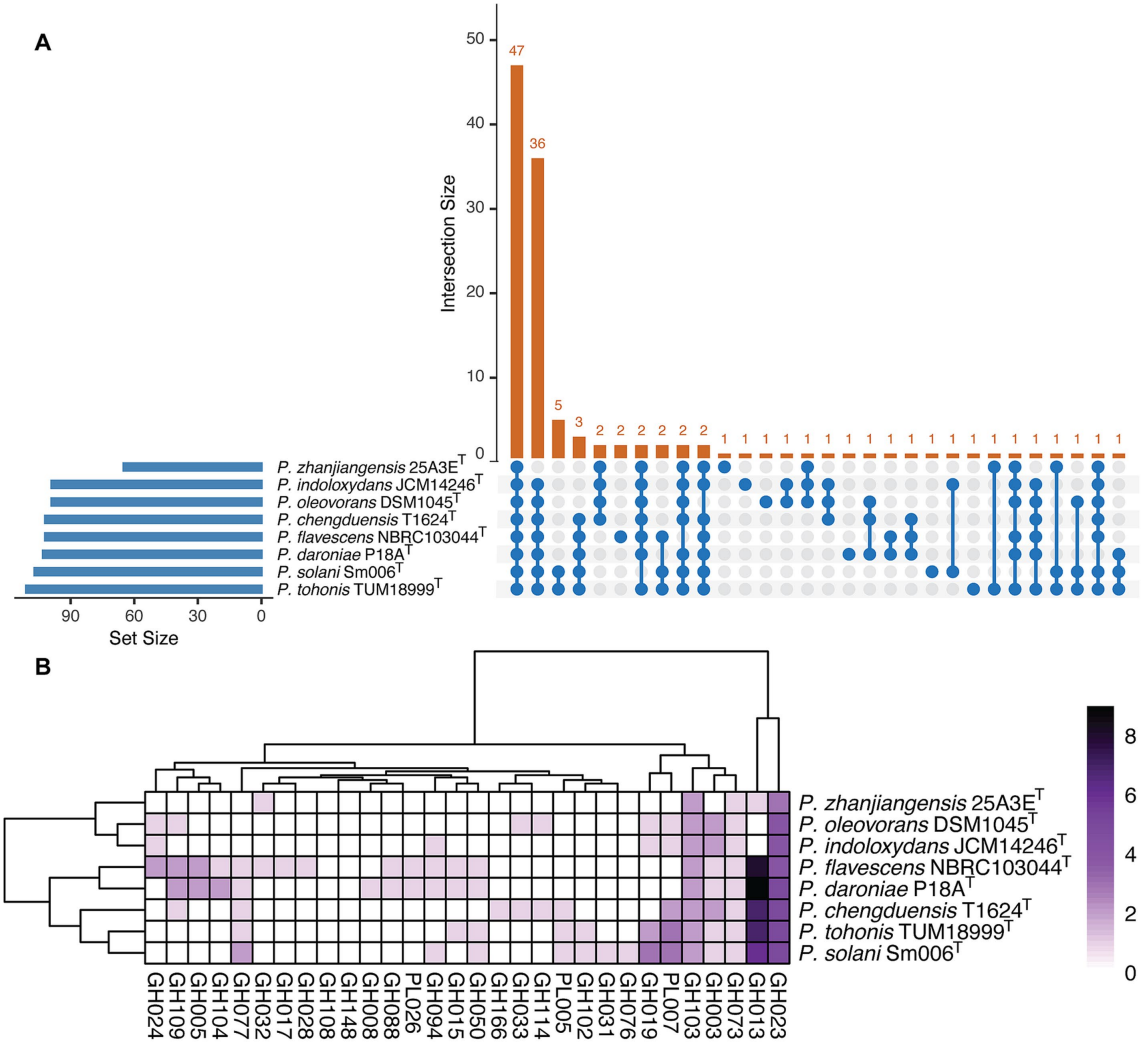
Metabolic capabilities analysis of the tested strains. **(A)** An Upset plot shows the intersections among KEGG module sets. The bar chart on the left displays the total number of KEGG modules identified for each strain, while the upper bar chart highlights the intersection size of KEGG modules shared across different strains. Blue connected dots in the bottom panel indicate which substrates are considered in each intersection. **(B)** Enzyme class distribution across all tested strains. Two specific enzyme classes are predicted among the eight genomes analyzed, with GH representing glycoside hydrolase and PL representing polysaccharide lyase.

The results of protein and pathway annotations generated by METABOLIC-G are summarized in Supplementary Table S3. Analysis showed that the eight genomic assemblies shared approximately 47% of identified KEGG modules (Figure 4A) and displayed similar profiles of carbohydrate-active enzymes (CAZymes) (Figure 4B), including polysaccharide lyases (PLs), glycoside hydrolases (GHs). These enzymes, involved in carbon metabolism, were closely linked to the COD removal ability of strain 25A3E^T^, enabling the degradation of carbohydrate-containing contaminants while simultaneously supplying energy for microbial metabolism.

**Figure 5:**
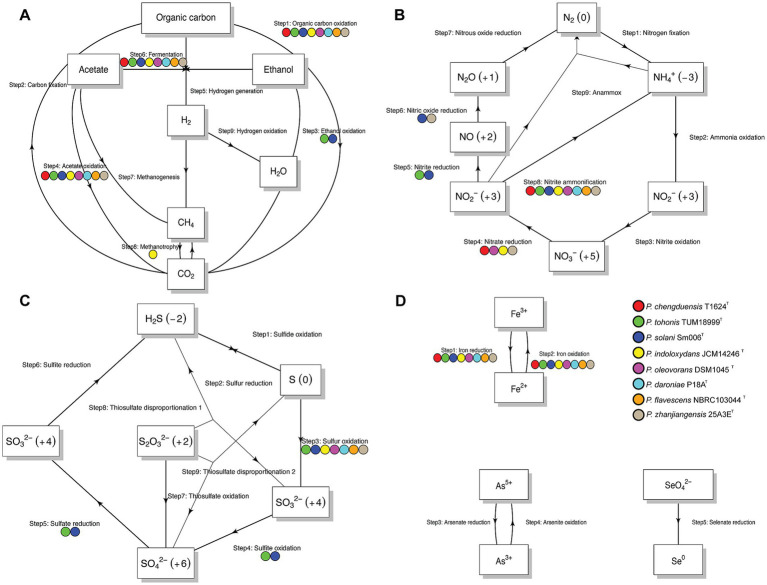
Summary diagram illustrates the biogeochemical cycling processes at the genomic level for each strain. **(A)** Carbon metabolism network; **(B)** Nitrogen metabolism network; **(C)** Sulfur metabolism network; **(D)** Other elements metabolism network. Each arrow corresponds to a specific transformation or step within the cycle. The labels above the arrows denote the step number and the associated reaction, with solid circles of different colors next to each arrow indicating the strains predicted to carry out that particular reaction.

The program also pinpointed specific pathways linked to energy metabolism and biogeochemistry within each genome, producing schematic representations of nitrogen, carbon, sulfur, and other elemental cycles (Figure 5). Our analysis found that all strains are capable of organic carbon oxidation and fermentation, acetate oxidation, nitrite ammonification, and iron oxidation and reduction. Notably, strain 25A3E^T^ was uniquely predicted to perform both nitrate reduction and nitric oxide reduction, distinguishing it from the other strains.

Among these strains, *P. chengduensis* WD211^T^, *P. chengduensis* BF6^T^, and *P. oleovorans* CT-WL5-6^T^ have been reported to have excellent wastewater treatment capacities. Wastewater treated with strain WD211^T^ showed concentration decreases of 89.39% in NH_4_^+^-N, 62.16% in NO_3_^−^, and 71.41% in COD after 24 h (Peng et al., 2023). Another study demonstrated that strain BF6^T^ could effectively remove nitrogen within 24 h under conditions of ammonia, nitrate, nitrite, and mixed nitrogen sources, with maximum removal efficiencies of total nitrogen reaching 97.00, 61.40, 79.10, and 84.98%, respectively (Yi et al., 2023). In a 96 h incubation of strain CT-WL5-6^T^ in alkaline media, approximately 90% of ammonia nitrogen was removed (Zhang et al., 2022). By contrast, strain 25A3E^T^ demonstrates the ability to degrade COD, NH_4_^+^-N, and TN in wastewater under low temperature conditions.

### 3.6 Genomic insights into low-temperature wastewater degradation

#### 3.6.1 Cold-adaptive genes

Cold-shock proteins (Csp) are produced by bacteria in response to a rapid decrease in temperature and play a crucial role in stabilizing DNA/RNA, thereby regulating transcription and translation processes under low-temperature conditions (Goordial et al., 2016; Keto-Timonen et al., 2016; Snopková et al., 2020). Strain 25A3E^T^ contains five Csp-coding genes, three for *cspA*, one for *cspC*, and one for *cspD* (Supplementary Table S4). Additionally, the genome of strain 25A3E^T^ harbors various genes involved in osmoregulation under low-temperature stress, such as those coding for glycine betaine transporters (*opuAB*, *opuAC*, *opuBB*, *opuBC*, and *opuD*) (Hoffmann and Bremer, 2011; Raiger Iustman et al., 2015). Ice-binding proteins (IBPs), which inhibit the growth of ice crystals inside and outside the cells, were also identified, with one gene copy of *ibp* found in strain 25A3E^T^ (Arai et al., 2019). Furthermore, the genome encodes a diverse array of enzymes and regulatory proteins that facilitate cold adaptation, including ribosome-binding factor A (*rbfA*), transcription termination protein A (*nusA*), translation initiation factors (*infA*, *infB*), RNA polymerase sigma factor (*rpoS*), cold adaptation genes (*deaD*), chaperone protein (*hscA*, *hscB*), and glutathione synthase (*gshB*) (Supplementary Table S4). In addition, the other cold adaptive genes were listed in Supplementary Table S4.

#### 3.6.2 Genes related to organic matter degradation

The COD value is an indicator of the organic matter content in water, and as shown in Figure 3, strain 25A3E^T^ exhibits strong capabilities in removing organic matter. Numerous oxidative enzymes, such as oxygenase, are critical in the degradation of organic substances (Kumari and Das, 2023). Genomic analysis of strain 25A3E^T^ revealed the presence of a large number of genes encoding monooxygenase (15 genes) and dioxygenases (34 genes) (Supplementary Table S5). These enzymes play a pivotal role in the oxidative degradation of organic compounds, contributing to the strain’s ability to reduce COD in wastewater.

#### 3.6.3 Genes related to nitrogen removal

Genomic analysis of strain 25A3E^T^ identified various genes related to nitrogen metabolism, which were essential for its nitrogen removal capabilities. Among these were nitrate/nitrite transport proteins (*nrtA*, *nrtB*, *nrtD*) responsible for transporting extracellular NO_3_^−^ and NO_2_^−^ into the cell. Assimilatory nitrate reductase (*nasA*) catalyzes the reduction of NO_3_^−^ to NO_2_^−^, which is subsequently reduced to NH₄^+^ by nitrite reductase (*nirB*, *nirD*). Additionally, genes encoding ammonium transporter, glutamate dehydrogenase (*gdhA*), glutamine synthetase (*glnA*), glutamate synthase (*gltB, gltD*), glutamate/aspartate transport (*gltI*), and carbamoyl-phosphate synthase (*carA, carB*) were found in the genomes of strain 25A3E^T^ (Supplementary Table S6). These enzymes facilitate a series of important biosynthetic reactions utilizing ammonia, speculating that NH_4_^+^-N is transformed into L-glutamine, which is then integrated into bacterial metabolism through glutamine pathways. In addition, the other nitrogen metabolism genes were listed in Supplementary Table S6.

### 3.7 Description of *Pseudomonas zhanjiangensis* sp. nov

*Pseudomonas zhanjiangensis* (zhan.jiang.en’sis. N.L. masc. Adj. *zhanjiangensis*, of zhanjiang, Guangdong province, China, where the type strain was isolated).

Cells of *Pseudomonas zhanjiangensis* sp. nov. are facultatively anaerobic, Gram-negative, motile, and rod-shaped, with dimensions of 0.75 μm in width and 1.5–1.75 μm in length. The strain exhibits growth at temperatures ranging from 4°C to 37°C, with an optimal growth temperature of 25°C. The pH range for growth is 6.0 to 10.0, with an optimal pH of 8.0. The strain tolerates NaCl concentrations from 0 to 8.0%, with optimal growth at 0.5% NaCl. The strain tests positive for both catalase and oxidase activities. It hydrolyzes starch and Tween 80 but does not hydrolyze cellulose, gelatin and Tween 20. According to the API 20NE test, the strain shows positive results for nitrate reduction to nitrites and esculin hydrolysis. It can assimilate glucose, maltose, gluconate, caprate, malate, and citrate. Weakly positive assimilation activities were observed for L-arabinose, D-mannose, and adipic acid. In the API ZYM test, the strain was positive for alkaline phosphatase, esterase (C4), esterase lipase (C8), valine arylamidase, and acid phosphatase, with weakly positive activities observed for lipase (C14) and naphthol-AS-BI-phosphohydrolase. Using Biolog GEN III MicroPlates, positive results were obtained for D-serine, L-alanine, L-arginine, L-aspartic acid, L-glutamic acid, L-histidine, L-serine, D-gluconic acid, L-lactic acid, citric acid, *α*-ketoglutaric acid, D-malic acid, L-malic acid, nalidixic acid, lithium chloride, potassium tellurite, *γ*-aminobutyric acid, propionic acid, acetic acid, and formic acid. The predominant polar lipids in strain 25A3E^T^ are sphingoglycolipid (SGL), and diphosphatidylglycerol (DPG). The major cellular fatty acids (>10%) are C_16:0_ (25.6%), C_17:0_ cyclo (12.2%), Summed feature 3 (16.7%), and Summed feature 8 (19.6%). The DNA G + C content of the strain is 65.4 mol%. The GenBank accession numbers for the 16S rRNA gene and the genome data of strain 25A3E^T^ are PP106243 and JBFTEG000000000, respectively. The type strain 25A3E^T^ (GDMCC 1.4380^T^ = JCM 36795^T^) was isolated from mangrove sediment in Zhanjiang, Guangdong Province, China.

## 4 Conclusion

*Pseudomonas zhanjiangensis* 25A3E^T^, isolated from mangrove sediment, has demonstrated a remarkable capacity for wastewater purification, particularly under low-temperature conditions. After 96 h at 10°C, wastewater treated with strain 25A3E^T^ showed a reduction of 72.9% in COD, 70.6% in NH_4_^+^-N, and 69.1% in TN. Genome analysis revealed the presence of genes associated with the removal of COD, NH_4_^+^-N, and TN, providing a genetic foundation for its functional capabilities. These findings underscore the strain’s significant potential for application in wastewater remediation.

## Data Availability

The datasets presented in this study can be found in online repositories. The names of the repository/repositories and accession number(s) can be found in the article/Supplementary material.

## Footnotes

^1^ www.ezbiocloud.net

^2^ http://sourceforge.net/projects/brig

^3^ http://ggdc.dsmz.de/distcalc2.php

^4^ https://github.com/zdf1987/EasyCGTree4

^5^ https://github.com/AnantharamanLab/METABOLIC

## References

[ref1] Ai C. Yan Z. Zhou H. Hou S. Chai L. Qiu G.. (2019). Metagenomic insights into the effects of seasonal temperature variation on the activities of activated sludge. Microorganisms 7:713. doi: 10.3390/microorganisms7120713, PMID: 31861224 PMC6956059

[ref2] Alikhan N.-F. Petty N. K. Ben Zakour N. L. Beatson S. A. (2011). BLAST ring image generator (BRIG): simple prokaryote genome comparisons. BMC Genomics 12, 1–10. doi: 10.1186/1471-2164-12-402 PMC3163573 21824423

[ref3] An Q. Deng S. Liu M. Li Z. Wu D. Wang T.. (2021). Study on the aerobic remediation of Ni (II) by *Pseudomonas hibiscicola* strain L1 interaction with nitrate. J. Environ. Manag. 299:113641. doi: 10.1016/j.jenvman.2021.113641, PMID: 34479150

[ref4] Anantharaman K. Brown C. T. Hug L. A. Sharon I. Castelle C. J. Probst A. J.. (2016). Thousands of microbial genomes shed light on interconnected biogeochemical processes in an aquifer system. Nat. Commun. 7:13219. doi: 10.1038/ncomms13219, PMID: 27774985 PMC5079060

[ref5] Arai T. Fukami D. Hoshino T. Kondo H. Tsuda S. (2019). Ice-binding proteins from the fungus *Antarctomyces psychrotrophicus* possibly originate from two different bacteria through horizontal gene transfer. FEBS J. 286, 946–962. doi: 10.1111/febs.14725, PMID: 30548092

[ref6] Aramaki T. Blanc-Mathieu R. Endo H. Ohkubo K. Kanehisa M. Goto S.. (2020). KofamKOALA: KEGG Ortholog assignment based on profile HMM and adaptive score threshold. Bioinformatics 36, 2251–2252. doi: 10.1093/bioinformatics/btz859, PMID: 31742321 PMC7141845

[ref7] Bankevich A. Nurk S. Antipov D. Gurevich A. A. Dvorkin M. Kulikov A. S.. (2012). SPAdes: a new genome assembly algorithm and its applications to single-cell sequencing. J. Comput. Biol. 19, 455–477. doi: 10.1089/cmb.2012.0021, PMID: 22506599 PMC3342519

[ref8] Basu N. B. Van Meter K. J. Byrnes D. K. Van Cappellen P. Brouwer R. Jacobsen B. H.. (2022). Managing nitrogen legacies to accelerate water quality improvement. Nat. Geosci. 15, 97–105. doi: 10.1038/s41561-021-00889-9

[ref9] Bueno-Gonzalez V. Brady C. Denman S. Plummer S. Allainguillaume J. Arnold D. (2019). *Pseudomonas daroniae* sp. nov. and *Pseudomonas dryadis* sp. nov., isolated from pedunculate oak affected by acute oak decline in the UK. Int. J. Syst. Evol. Microbiol. 69, 3368–3376. doi: 10.1099/ijsem.0.003615 31391144

[ref10] Carlier A. Beaumel M. Moreau S. Acar T. Sana T. G. Cnockaert M.. (2024). *Pseudomonas fortuita* sp. nov., isolated from the endosphere of a wild yam. Int. J. Syst. Evol. Microbiol. 44:006395, 410–415. doi: 10.1099/ijsem.0.006395 38940814

[ref11] Cavicchioli R. Siddiqui K. S. Andrews D. Sowers K. R. (2002). Low-temperature extremophiles and their applications. Curr. Opin. Biotechnol. 13, 253–261. doi: 10.1016/s0958-1669(02)00317-8 12180102

[ref12] Chun J. Oren A. Ventosa A. Christensen H. Arahal D. R. da Costa M. S.. (2018). Proposed minimal standards for the use of genome data for the taxonomy of prokaryotes. Int. J. Syst. Evol. Microbiol. 68, 461–466. doi: 10.1099/ijsem.0.002516, PMID: 29292687

[ref13] Collins M. Jones D. (1980). Lipids in the classification and identification of coryneform bacteria containing peptidoglycans based on 2, 4-diaminobutyric acid. J. Appl. Microbiol. 48, 459–470. doi: 10.1111/j.1365-2672.1980.tb01036.x

[ref14] Duke N. C. Meynecke J.-O. Dittmann S. Ellison A. M. Anger K. Berger U.. (2007). A world without mangroves? Science 317, 41–42. doi: 10.1126/science.317.5834.41b 17615322

[ref15] Fan Y. Zhou Z. Liu F. Qian L. Yu X. Huang F.. (2024). The vertical partitioning between denitrification and dissimilatory nitrate reduction to ammonium of coastal mangrove sediment microbiomes. Water Res. 262:122113. doi: 10.1016/j.watres.2024.122113, PMID: 39032335

[ref16] Felsenstein J. (1981). Evolutionary trees from DNA sequences: a maximum likelihood approach. J. Mol. Evol. 17, 368–376. doi: 10.1007/BF01734359 7288891

[ref17] Finn R. D. Tate J. Mistry J. Coggill P. C. Sammut S. J. Hotz H.-R.. (2008). The Pfam protein families database. Nucleic Acids Res. 36, D281–D288. doi: 10.1093/nar/gkm960, PMID: 18039703 PMC2238907

[ref18] Fykse E. M. Tjärnhage T. Humppi T. Eggen V. S. Ingebretsen A. Skogan G.. (2015). Identification of airborne bacteria by 16S rDNA sequencing, MALDI-TOF MS and the MIDI microbial identification system. Aerobiologia 31, 271–281. doi: 10.1007/s10453-015-9363-9, PMID: 32214629 PMC7087874

[ref19] Ge H.-Y. Zhang Y.-H. Hu Y.-Q. Li H.-R. Han W. du Y.. (2024). *Pseudomonas paeninsulae* sp. nov. and *Pseudomonas svalbardensis* sp. nov., isolated from Antarctic intertidal sediment and Arctic soil, respectively. Int. J. Syst. Evol. Microbiol. 74:006466. doi: 10.1099/ijsem.0.006466, PMID: 39073408

[ref20] Goordial J. Raymond-Bouchard I. Zolotarov Y. De Bethencourt L. Ronholm J. Shapiro N.. (2016). Cold adaptive traits revealed by comparative genomic analysis of the eurypsychrophile *Rhodococcus* sp. JG3 isolated from high elevation McMurdo Dry Valley permafrost, Antarctica. FEMS Microbiol. Ecol. 92:fiv154. doi: 10.1093/femsec/fiv154, PMID: 26637477

[ref21] Goris J. Konstantinidis K. T. Klappenbach J. A. Coenye T. Vandamme P. Tiedje J. M. (2007). DNA–DNA hybridization values and their relationship to whole-genome sequence similarities. Int. J. Syst. Evol. Microbiol. 57, 81–91. doi: 10.1099/ijs.0.64483-0 17220447

[ref22] Gross H. Loper J. E. (2009). Genomics of secondary metabolite production by *Pseudomonas* spp. Nat. Prod. Rep. 26, 1408–1446. doi: 10.1039/b817075b 19844639

[ref23] Guo Y. Wang Y. Zhang Z. Huang F. Chen S. (2018). Physiological and transcriptomic insights into the cold adaptation mechanism of a novel heterotrophic nitrifying and aerobic denitrifying-like bacterium *Pseudomonas indoloxydans* YY-1. Int. Biodeterior. Biodegrad. 134, 16–24. doi: 10.1016/j.ibiod.2018.08.001

[ref24] Hildebrand D. Palleroni N. Hendson M. Toth J. Johnson J. (1994). *Pseudomonas flavescens* sp. nov., isolated from walnut blight cankers. Int. J. Syst. Evol. Microbiol. 44, 410–415. doi: 10.1099/00207713-44-3-410, PMID: 7520732

[ref25] Hoffmann T. Bremer E. (2011). Protection of *Bacillus subtilis* against cold stress via compatible-solute acquisition. J. Bacteriol. 193, 1552–1562. doi: 10.1128/JB.01319-10, PMID: 21296969 PMC3067655

[ref26] Hou N. Yang X. Wang W. Sardans J. Yin X. Jiang F.. (2024). Mangrove wetland recovery enhances soil carbon sequestration capacity of soil aggregates and microbial network stability in southeastern China. Sci. Total Environ. 951:175586. doi: 10.1016/j.scitotenv.2024.175586, PMID: 39154998

[ref27] Hu W. Li Z. Ou H. Wang X. Wang Q. Tao Z.. (2023). Novosphingobium album sp. nov., Novosphingobium organovorum sp. nov. and Novosphingobium mangrovi sp. nov. with the organophosphorus pesticides degrading ability isolated from mangrove sediments. Int. J. Syst. Evol. Microbiol. 73:005843. doi: 10.1099/ijsem.0.005843, PMID: 37115596

[ref28] Huang J. Ai G. Liu N. Huang Y. (2022). Environmental adaptability and organic pollutant degradation capacity of a novel *Rhodococcus* species derived from soil in the uninhabited area of the Qinghai-Tibet plateau. Microorganisms 10:1935. doi: 10.3390/microorganisms10101935, PMID: 36296211 PMC9609184

[ref29] Huang T. Guo L. Zhang H. Su J. Wen G. Zhang K. (2015). Nitrogen-removal efficiency of a novel aerobic denitrifying bacterium, *Pseudomonas stutzeri* strain ZF31, isolated from a drinking-water reservoir. Bioresour. Technol. 196, 209–216. doi: 10.1016/j.biortech.2015.07.059, PMID: 26241840

[ref30] Hyatt D. Chen G.-L. LoCascio P. F. Land M. L. Larimer F. W. Hauser L. J. (2010). Prodigal: prokaryotic gene recognition and translation initiation site identification. BMC Bioinf. 11, 1–11. doi: 10.1186/1471-2105-11-119, PMID: 20211023 PMC2848648

[ref31] Kanehisa M. Goto S. (2000). KEGG: Kyoto encyclopedia of genes and genomes. Nucleic Acids Res. 28, 27–30. doi: 10.1093/nar/28.1.27, PMID: 10592173 PMC102409

[ref32] Keto-Timonen R. Hietala N. Palonen E. Hakakorpi A. Lindström M. Korkeala H. (2016). Cold shock proteins: a minireview with special emphasis on Csp-family of enteropathogenic Yersinia. Front. Microbiol. 7:1151. doi: 10.3389/fmicb.2016.01151, PMID: 27499753 PMC4956666

[ref33] Khin T. Annachhatre A. P. (2004). Novel microbial nitrogen removal processes. Biotechnol. Adv. 22, 519–532. doi: 10.1016/j.biotechadv.2004.04.003 15262315

[ref34] Kimura M. (1980). A simple method for estimating evolutionary rates of base substitutions through comparative studies of nucleotide sequences. J. Mol. Evol. 16, 111–120. doi: 10.1007/BF01731581 7463489

[ref35] Kitahara K. Yasutake Y. Miyazaki K. (2012). Mutational robustness of 16S ribosomal RNA, shown by experimental horizontal gene transfer in *Escherichia coli*. Proc. Natl. Acad. Sci. 109, 19220–19225. doi: 10.1073/pnas.1213609109, PMID: 23112186 PMC3511107

[ref36] Kumar S. Stecher G. Li M. Knyaz C. Tamura K. (2018). MEGA X: molecular evolutionary genetics analysis across computing platforms. Mol. Biol. Evol. 35, 1547–1549. doi: 10.1093/molbev/msy096 29722887 PMC5967553

[ref37] Kumari S. Das S. (2023). Bacterial enzymatic degradation of recalcitrant organic pollutants: catabolic pathways and genetic regulations. Environ. Sci. Pollut. Res. 30, 79676–79705. doi: 10.1007/s11356-023-28130-7, PMID: 37330441

[ref38] Larkin M. A. Blackshields G. Brown N. P. Chenna R. McGettigan P. A. McWilliam H.. (2007). Clustal W and Clustal X version 2.0. Bioinformatics 23, 2947–2948. doi: 10.1093/bioinformatics/btm404 17846036

[ref39] Lee I. Ouk Kim Y. Park S.-C. Chun J. (2016). OrthoANI: an improved algorithm and software for calculating average nucleotide identity. Int. J. Syst. Evol. Microbiol. 66, 1100–1103. doi: 10.1099/ijsem.0.000760, PMID: 26585518

[ref40] Lefebvre O. Moletta R. (2006). Treatment of organic pollution in industrial saline wastewater: a literature review. Water Res. 40, 3671–3682. doi: 10.1016/j.watres.2006.08.027 17070895

[ref41] Lei Y. Wang Y. Liu H. Xi C. Song L. (2016). A novel heterotrophic nitrifying and aerobic denitrifying bacterium, *Zobellella taiwanensis* DN-7, can remove high-strength ammonium. Appl. Microbiol. Biotechnol. 100, 4219–4229. doi: 10.1007/s00253-016-7290-5, PMID: 26762390

[ref42] Li C. Yang J. Wang X. Wang E. Li B. He R.. (2015). Removal of nitrogen by heterotrophic nitrification–aerobic denitrification of a phosphate accumulating bacterium *Pseudomonas stutzeri* YG-24. Bioresour. Technol. 182, 18–25. doi: 10.1016/j.biortech.2015.01.100 25668754

[ref43] Lick S. Wibberg D. Busche T. Blom J. Grimmler C. Goesmann A.. (2024). *Pseudomonas kulmbachensis* sp. nov. and Pseudomonas paraveronii sp. nov., originating from chilled beef and chicken breast. Int. J. Syst. Evol. Microbiol. 74:006293. doi: 10.1099/ijsem.0.006293, PMID: 38587505

[ref44] Lin Z. Huang W. Zhou J. He X. Wang J. Wang X.. (2020). The variation on nitrogen removal mechanisms and the succession of ammonia oxidizing archaea and ammonia oxidizing bacteria with temperature in biofilm reactors treating saline wastewater. Bioresour. Technol. 314:123760. doi: 10.1016/j.biortech.2020.123760, PMID: 32634643

[ref45] Manickam N. Ghosh A. Jain R. K. Mayilraj S. (2008). Description of a novel indole-oxidizing bacterium *Pseudomonas indoloxydans* sp. nov., isolated from a pesticide-contaminated site. Syst. Appl. Microbiol. 31, 101–107. doi: 10.1016/j.syapm.2008.02.002, PMID: 18406094

[ref46] Marasco R. Michoud G. Sefrji F. O. Fusi M. Antony C. P. Seferji K. A.. (2023). The identification of the new species *Nitratireductor thuwali* sp. nov. reveals the untapped diversity of hydrocarbon-degrading culturable bacteria from the arid mangrove sediments of the Red Sea. Front. Microbiol. 14:1155381. doi: 10.3389/fmicb.2023.1155381 37200916 PMC10185800

[ref47] Meier-Kolthoff J. P. Auch A. F. Klenk H.-P. Göker M. (2013). Genome sequence-based species delimitation with confidence intervals and improved distance functions. BMC Bioinf. 14, 1–14. doi: 10.1186/1471-2105-14-60, PMID: 23432962 PMC3665452

[ref48] Minnikin D. Collins M. Goodfellow M. (1979). Fatty acid and polar lipid composition in the classification of Cellulomonas, Oerskovia and related taxa. J. Appl. Microbiol. 47, 87–95. doi: 10.1111/j.1365-2672.1979.tb01172.x

[ref49] Mukherji S. Bakshi U. Ghosh A. (2022). Draft genome sequences of hydrocarbon degrading Haloferax sp. AB510, Haladaptatus sp. AB618 and Haladaptatus sp. AB643 isolated from the estuarine sediments of Sundarban mangrove forests, India. 3 Biotech 12:204. doi: 10.1007/s13205-022-03273-5, PMID: 35935548 PMC9349328

[ref50] Naruya Saitou M. N. (1987). The neighbor-joining method: a new method for reconstructing phylogenetic trees. Mol. Biol. Evol. 4, 406–425. doi: 10.1093/oxfordjournals.molbev.a040454 3447015

[ref51] Nicklasson M. Martín-Rodríguez A. J. Thorell K. Higdon S. M. Neves L. Mussagy A.. (2022). *Pseudomonas boanensis* sp. nov., a bacterium isolated from river water used for household purposes in Boane District, Mozambique. Int. J. Syst. Evol. Microbiol. 72:005461. doi: 10.1099/ijsem.0.005461, PMID: 35819404

[ref52] Pardi F. Guillemot S. Gascuel O. (2010). Robustness of phylogenetic inference based on minimum evolution. Bull. Math. Biol. 72, 1820–1839. doi: 10.1007/s11538-010-9510-y 20449671

[ref53] Parks D. H. Imelfort M. Skennerton C. T. Hugenholtz P. Tyson G. W. (2015). CheckM: assessing the quality of microbial genomes recovered from isolates, single cells, and metagenomes. Genome Res. 25, 1043–1055. doi: 10.1101/gr.186072.114, PMID: 25977477 PMC4484387

[ref54] Parks D. H. Rinke C. Chuvochina M. Chaumeil P.-A. Woodcroft B. J. Evans P. N.. (2017). Recovery of nearly 8,000 metagenome-assembled genomes substantially expands the tree of life. Nat. Microbiol. 2, 1533–1542. doi: 10.1038/s41564-017-0012-7, PMID: 28894102

[ref55] Peng H. Wu H. Gu W. Lu Y. Qin H. You Y.. (2023). Exploring the application potential of aquaculture sewage treatment of *Pseudomonas chengduensis* strain WD211 based on its complete genome. Genes 14:2107. doi: 10.3390/genes14122107, PMID: 38136929 PMC10743257

[ref56] Raiger Iustman L. J. Tribelli P. M. Ibarra J. G. Catone M. V. Solar Venero E. C. López N. I. (2015). Genome sequence analysis of *Pseudomonas extremaustralis* provides new insights into environmental adaptability and extreme conditions resistance. Extremophiles 19, 207–220. doi: 10.1007/s00792-014-0700-7, PMID: 25316211

[ref57] Saha R. Spröer C. Beck B. Bagley S. (2010). *Pseudomonas oleovorans* subsp. lubricantis subsp. nov., and reclassification of *Pseudomonas pseudoalcaligenes* ATCC 17440^T^ as later synonym of *Pseudomonas oleovorans* ATCC 8062^T^. Curr. Microbiol. 60, 294–300. doi: 10.1007/s00284-009-9540-6, PMID: 19936829

[ref58] Sawada H. Takeuchi K. Someya N. Morohoshi T. Satou M. (2023). *Pseudomonas solani* sp. nov. isolated from the rhizosphere of eggplant in Japan. Int. J. Syst. Evol. Microbiol. 73:005942. doi: 10.1099/ijsem.0.005942, PMID: 37347683

[ref59] Selengut J. D. Haft D. H. Davidsen T. Ganapathy A. Gwinn-Giglio M. Nelson W. C.. (2007). TIGRFAMs and genome properties: tools for the assignment of molecular function and biological process in prokaryotic genomes. Nucleic Acids Res. 35, D260–D264. doi: 10.1093/nar/gkl1043, PMID: 17151080 PMC1781115

[ref60] Shahid M. J. Ali S. Shabir G. Siddique M. Rizwan M. Seleiman M. F.. (2020). Comparing the performance of four macrophytes in bacterial assisted floating treatment wetlands for the removal of trace metals (Fe, Mn, Ni, Pb, and Cr) from polluted river water. Chemosphere 243:125353. doi: 10.1016/j.chemosphere.2019.125353, PMID: 31765899

[ref61] Sheaves M. (2009). Consequences of ecological connectivity: the coastal ecosystem mosaic. Mar. Ecol. Prog. Ser. 391, 107–115. doi: 10.3354/meps08121

[ref62] Silby M. W. Winstanley C. Godfrey S. A. Levy S. B. Jackson R. W. (2011). *Pseudomonas* genomes: diverse and adaptable. FEMS Microbiol. Rev. 35, 652–680. doi: 10.1111/j.1574-6976.2011.00269.x 21361996

[ref63] Snopková K. Čejková D. Dufková K. Sedláček I. Šmajs D. (2020). Genome sequences of two Antarctic strains of *Pseudomonas prosekii*: insights into adaptation to extreme conditions. Arch. Microbiol. 202, 447–454. doi: 10.1007/s00203-019-01755-4, PMID: 31691844

[ref64] Tao Y. Zhou Y. He X. Hu X. Li D. (2014). *Pseudomonas chengduensis* sp. nov., isolated from landfill leachate. Int. J. Syst. Evol. Microbiol. 64, 95–100. doi: 10.1099/ijs.0.050294-0, PMID: 24021726

[ref65] Thatoi H. Behera B. C. Mishra R. R. Dutta S. K. (2013). Biodiversity and biotechnological potential of microorganisms from mangrove ecosystems: a review. Ann. Microbiol. 63, 1–19. doi: 10.1007/s13213-012-0442-7

[ref66] Tindall B. J. Sikorski J. Smibert R. A. Krieg N. R. (2014). Phenotypic characterization and the principles of comparative systematics. Methods Gen. Mol. Microbiol. 15, 330–393. doi: 10.1128/9781555817497.ch15

[ref67] Tong Y. Li Y. Qin W. Wu S. Xu W. Jin P.. (2023). New insight into the metabolic mechanism of a novel lipid-utilizing and denitrifying bacterium capable of simultaneous removal of nitrogen and grease through transcriptome analysis. Front. Microbiol. 14:1258003. doi: 10.3389/fmicb.2023.1258003, PMID: 37965562 PMC10642853

[ref68] Wei-dong H. (2003). Present status and conservation strategies of mangrove resource in Guangdong, P.R. China. J. For. Res. 14, 151–154. doi: 10.1007/BF02856783

[ref69] Wu X. Zhou H. Li L. Wang E. Zhou X. Gu Y.. (2020). Whole genome sequencing and comparative genomic analyses of *Lysinibacillus pakistanensis* LZH-9, a halotolerant strain with excellent COD removal capability. Microorganisms 8:716. doi: 10.3390/microorganisms8050716, PMID: 32408484 PMC7284689

[ref70] Xie C.-J. Yao L. Tang R. Han S. Yang S. Alwathnani H.. (2024). *Azotosporobacter soli* gen. Nov., sp. nov., a novel nitrogen-fixing bacterium isolated from paddy soil. Antonie Van Leeuwenhoek 117, 79–10. doi: 10.1007/s10482-024-01978-6, PMID: 38755437

[ref71] Xu Z. Ben Y. Chen Z. Jiang A. Shen J. Han X. (2018). Application and microbial ecology of psychrotrophs in domestic wastewater treatment at low temperature. Chemosphere 191, 946–953. doi: 10.1016/j.chemosphere.2017.10.121 29145139

[ref72] Yamada K. Sasaki M. Aoki K. Nagasawa T. Murakami H. Ishii M.. (2021). *Pseudomonas tohonis* sp. nov., isolated from the skin of a patient with burn wounds in Japan. Int. J. Syst. Evol. Microbiol. 71:005115. doi: 10.1099/ijsem.0.005115, PMID: 34762579

[ref73] Ye Y. Yan C. Nie Y. Zhang J. Zhao Z. Zhang R.. (2020). *Nitratireductor mangrovi* sp. nov., a nitrate-reducing bacterium isolated from mangrove soil. Curr. Microbiol. 77, 1334–1340. doi: 10.1007/s00284-020-01885-9, PMID: 32123982

[ref74] Yi M. Wang H. Ma X. Wang C. Wang M. Liu Z.. (2023). Efficient nitrogen removal of a novel *Pseudomonas chengduensis* strain BF6 mainly through assimilation in the recirculating aquaculture systems. Bioresour. Technol. 379:129036. doi: 10.1016/j.biortech.2023.129036, PMID: 37037330

[ref75] Yoon S.-H. Ha S.-M. Kwon S. Lim J. Kim Y. Seo H.. (2017). Introducing EzBioCloud: a taxonomically united database of 16S rRNA gene sequences and whole-genome assemblies. Int. J. Syst. Evol. Microbiol. 67, 1613–1617. doi: 10.1099/ijsem.0.001755, PMID: 28005526 PMC5563544

[ref76] Zhang D.-F. He W. Shao Z. Ahmed I. Zhang Y. Li W.-J.. (2023a). EasyCGTree: a pipeline for prokaryotic phylogenomic analysis based on core gene sets. Bioinformatics 24:390. doi: 10.1186/s12859-023-05527-2, PMID: 37838689 PMC10576351

[ref77] Zhang R. Luo L. Wang S. Guo K. Xu W. Zhao Z. (2022). Screening and characteristics of ammonia nitrogen removal bacteria under alkaline environments. Front. Microbiol. 13:969722. doi: 10.3389/fmicb.2022.969722, PMID: 36081787 PMC9444525

[ref78] Zhang X. Shi H.-T. Feng X.-C. Jiang C.-Y. Wang W.-Q. Xiao Z.-J.. (2023b). Efficient aerobic denitrification without nitrite accumulation by *Pseudomonas mendocina* HITSZ-D1 isolated from sewage sludge. Bioresour. Technol. 379:129039. doi: 10.1016/j.biortech.2023.129039, PMID: 37037332

[ref79] Zhou Z. Tran P. Q. Breister A. M. Liu Y. Kieft K. Cowley E. S.. (2022). METABOLIC: high-throughput profiling of microbial genomes for functional traits, metabolism, biogeochemistry, and community-scale functional networks. Microbiome 10:33. doi: 10.1186/s40168-021-01213-8, PMID: 35172890 PMC8851854

[ref80] Zhou J. Zhang C.-J. Li M. (2023). *Desulfovibrio mangrovi* sp. nov., a sulfate-reducing bacterium isolated from mangrove sediments: a member of the proposed genus “*Psychrodesulfovibrio*”. Antonie Van Leeuwenhoek 116, 499–510. doi: 10.1007/s10482-023-01820-5 36917346

