# Supplementary material for: Efficient low-temperature wastewater treatment by Pseudomonas zhanjiangensis sp. nov.: a novel cold-tolerant bacterium isolated from mangrove sediment
Source: Front Microbiol. 2024 Oct 31;15:1491174. doi: 10.3389/fmicb.2024.1491174 (PMC11560893; doi:10.3389/fmicb.2024.1491174)
Supplement: Supplementary file 1 [file Data_Sheet_1.zip › Data Sheet 1/Supplementary Figures (S1-S8) and Tables (S1-S2).DOCX]

Supplementary Material

# Supplementary Figures and Tables

## Supplementary Figures


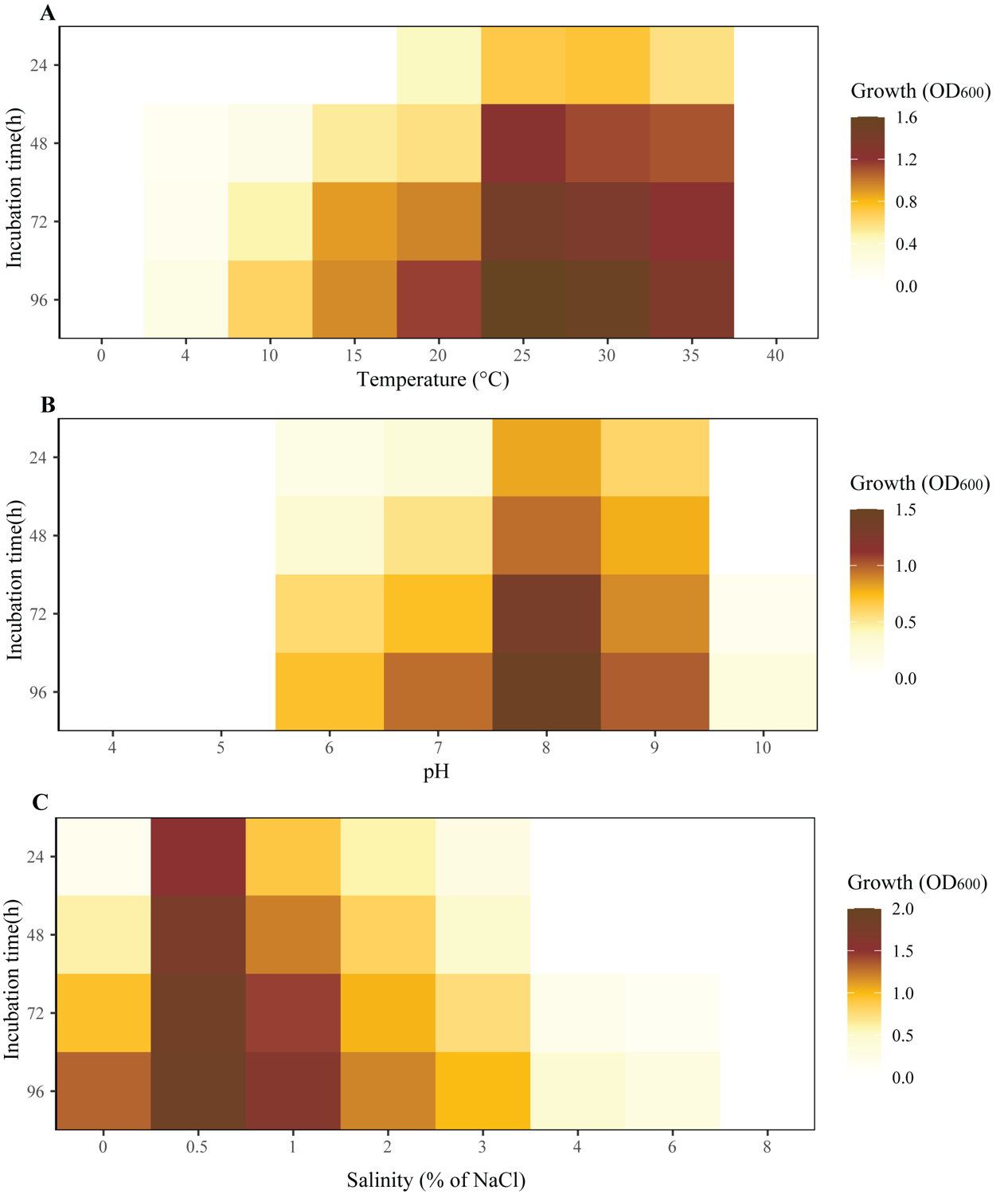


**Supplementary Figure 1.** Heatmaps illustrating the growth of strain 25A3E^T^ under various conditions, as indicated by mean OD_600_ values. The panels show growth at different (A) temperatures, (B) pH levels, and (C) salinity levels (% NaCl).


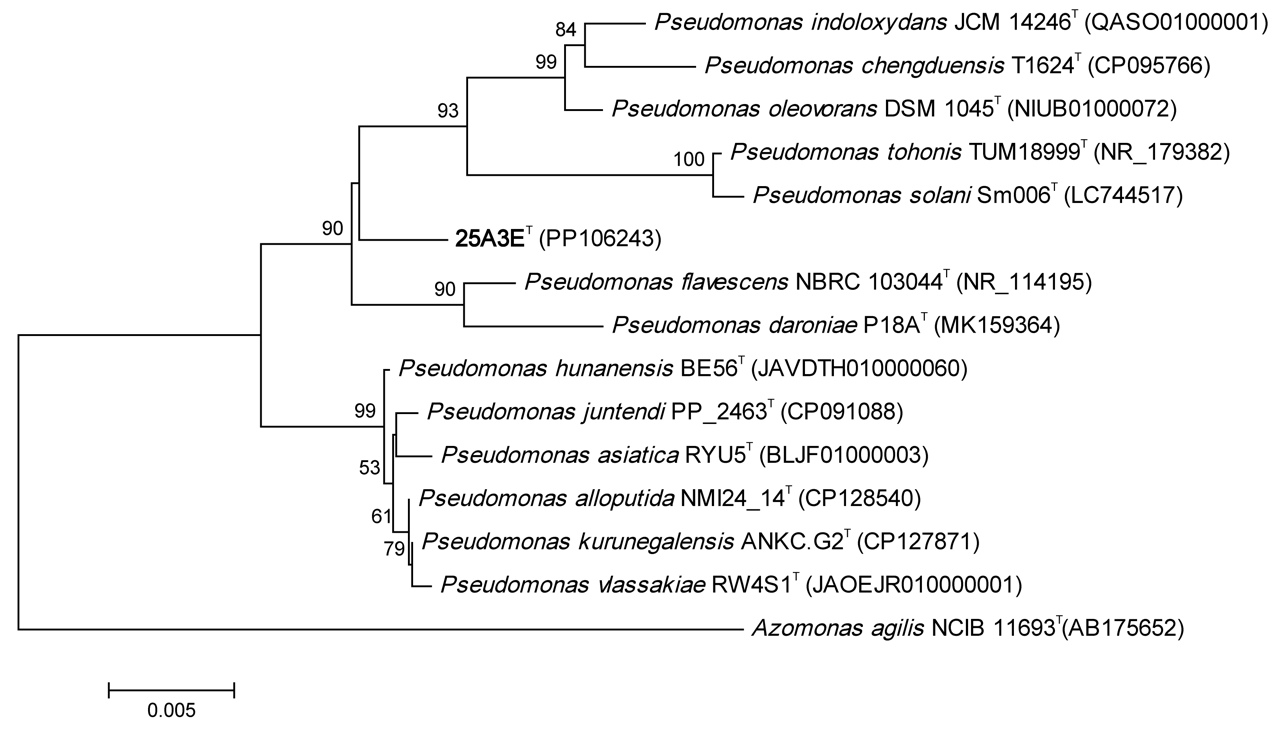


**Supplementary Figure 2.** Neighbor-joining tree based on 16S rRNA gene sequences showing the phylogenetic relationships between strain 25A3E^T^ and its closely related species. Bootstrap percentages (based on 1000 replications) above 50% are shown at the nodes. GenBank accession numbers are provided in parentheses, Bar, 0.005 substitutions per nucleotide position.


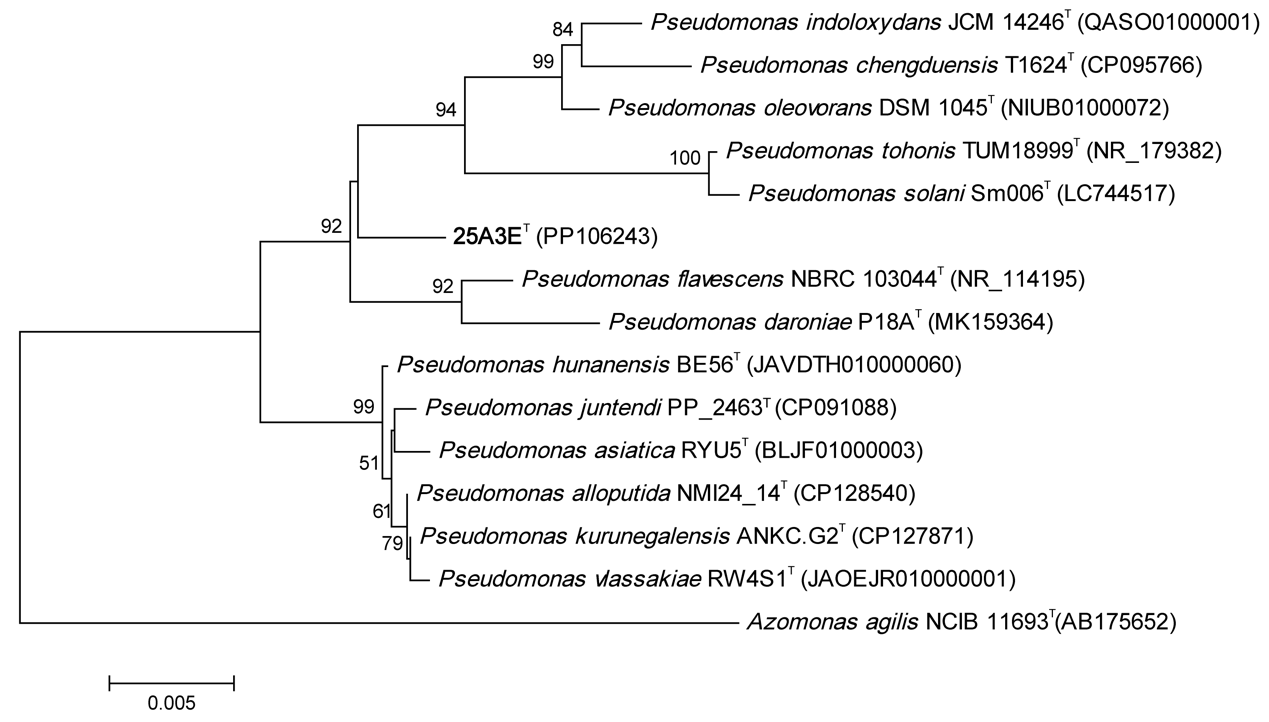


**Supplementary Figure 3.** Minimum-evolution phylogenetic tree based on 16S rRNA gene sequence. Bootstrap values (expressed as percentages of 1000 replications) greater than 50% are shown at branch points. Bar, 0.005 substitutions per nucleotide position.


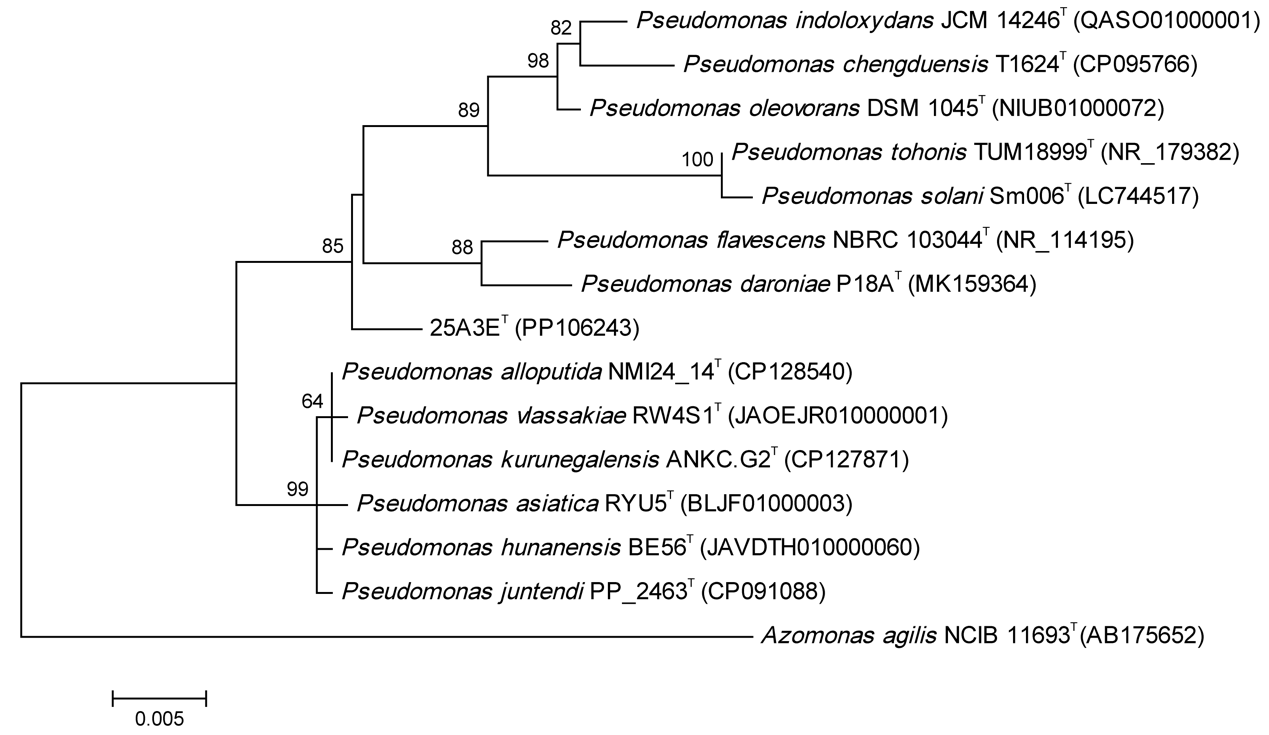


**Supplementary Figure 4.** Maximum-likelihood phylogenetic tree based on 16S rRNA gene sequence. Bootstrap values (expressed as percentages of 1000 replications) greater than 50% are shown at branch points. Bar, 0.005 substitutions per nucleotide position.

**
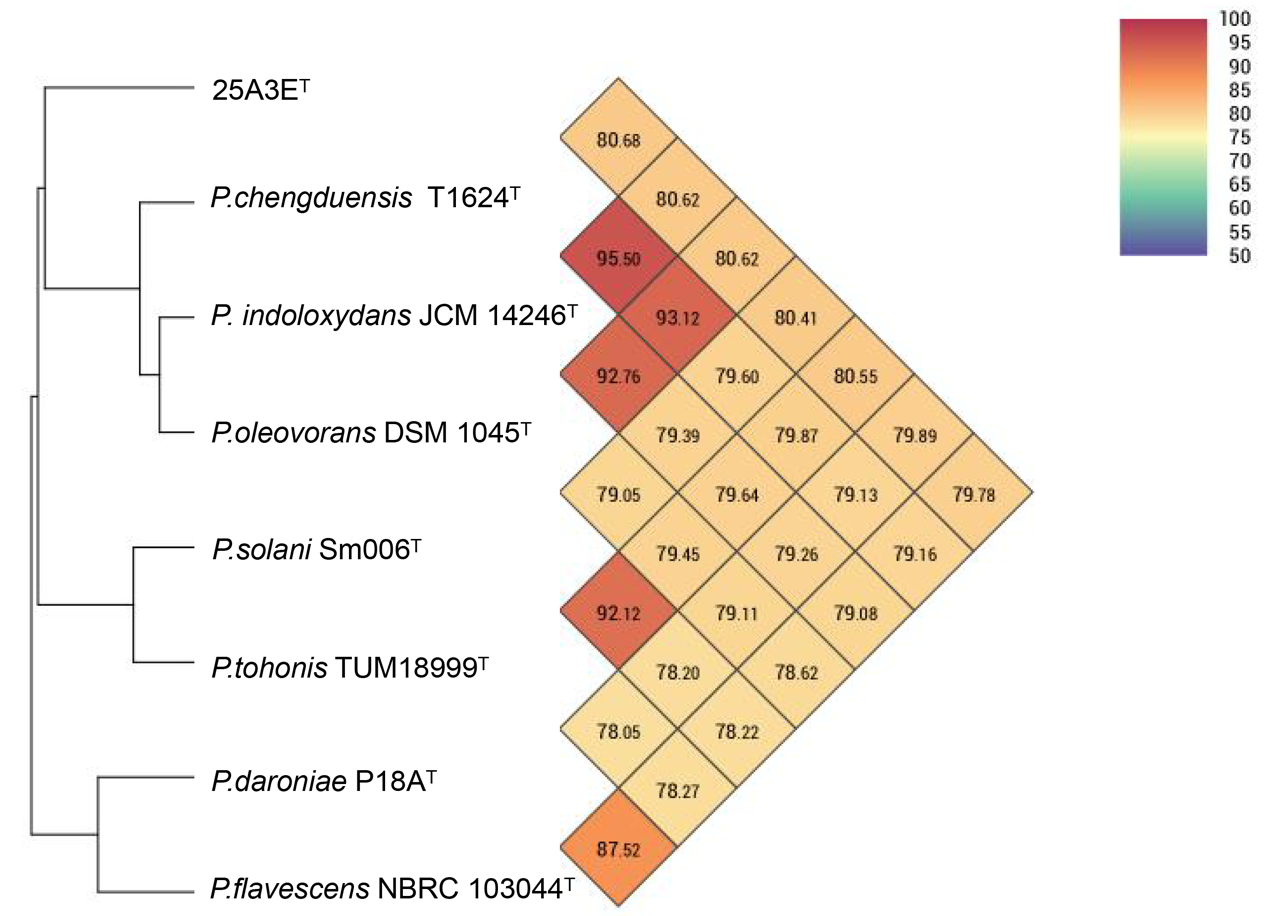
**

**Supplementary Figure 5.** Heatmap (OrthoANI) indicating the genetic relatedness between 25A3E^T^ and closely related taxa displayed as a cladogram.

**
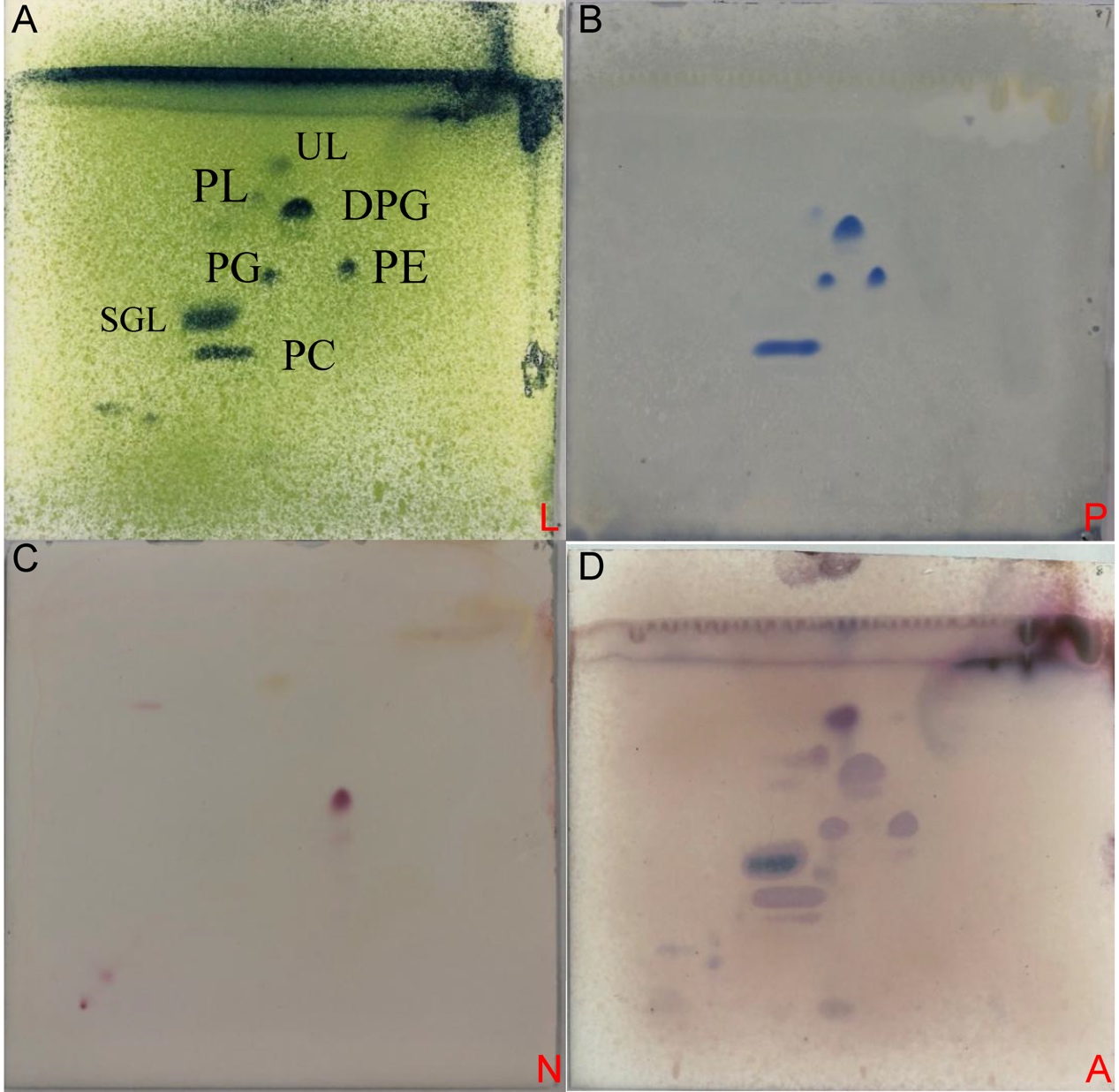
**

**Supplementary Figure 6.** Two-dimensional thin-layer chromatography analysis of polar lipids from strain 25A3E^T^. Abbreviations: DPG, diphosphatidylglycerol; PG, phosphatidylglycerol; PL, phospholipid; PE, phosphatidylethanolamine; PC, Phosphatidylcholine; UL, unidentified lipids; SGL, sphingoglycolipid; L, molybdatophosphoric acid reagent; P, phosphate stain reagent; N, ninhydrin stain reagent; A, anisaldehyde stain reagent.


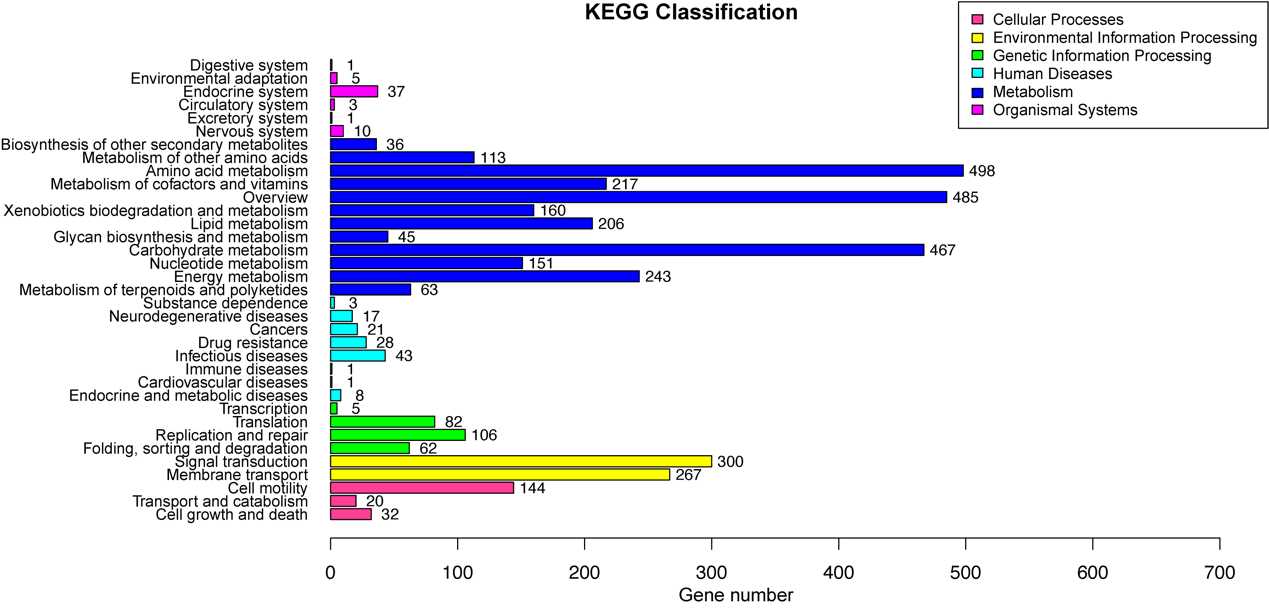


**Supplementary Figure 7.** Numbers of genes associated with KEGG functional categories of strain 25A3E^T^.


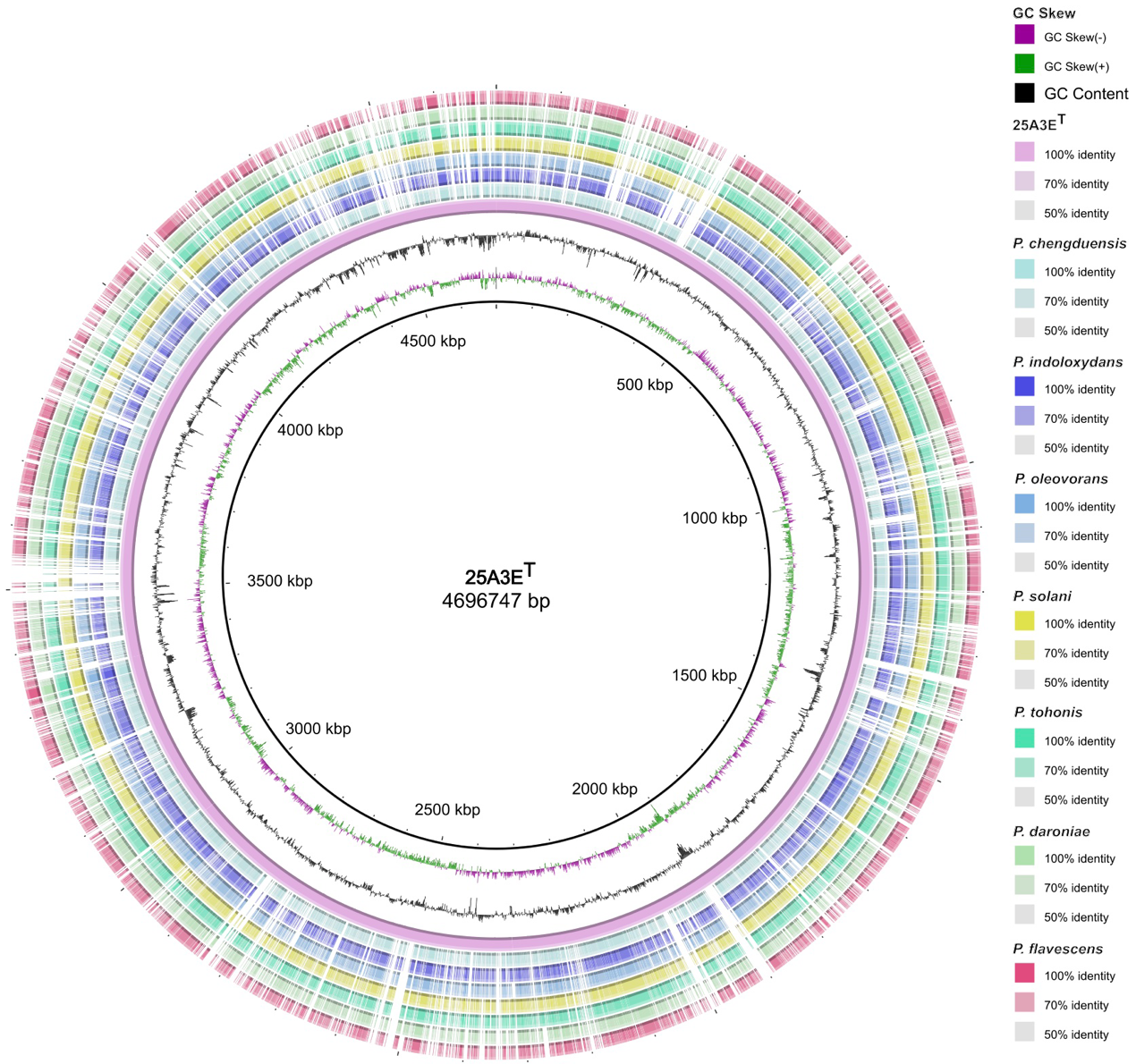


**Supplementary Figure 8.** Circular map representing a BLASTn comparison of the genomes of strain 25A3E^T^ and its closely related species. From inner to outer: Ring 1, GC% content; Ring 2, G + C positive skew (green), G + C negative skew (purple); Ring 3, 25A3E^T^; Ring 4, *P. chengduensis* T1624^T^; Ring 5, *P. indoloxydans* JCM 14246^T^; Ring 6, *P. oleovorans* DSM 1045^T^; Ring 7, *P. solani* Sm006^T^ ; Ring 8, *P. tohonis* TUM18999^T^; Ring 9, *P. daroniae* P18A^T^ ; Ring 10, *P. flavescens* NBRC103044^T^.

## Supplementary Tables

**Supplementary Table 1.** Cellular fatty acid contents (>0.5%) of strain 25A3E^T^ and related species.

| Fatty acid | 1 | 2 | 3 | 4 | 5 | 6 | 7 | 8 |
| --- | --- | --- | --- | --- | --- | --- | --- | --- |
| C_10:0_ 3-OH | 6.6 | 3.13 | 3.08 | 4.1 | 3.7 | 3.14 | 3.3 | 3.7 |
| C_12:0_ 3-OH | 4.9 | 3.81 | 3.93 | 4.1 | 4.2 | 4.26 | 3.8 | 3.6 |
| C_12:0_ | 6.4 | 7.46 | 7.74 | 10.5 | 8.5 | 8.25 | 9.9 | 9.2 |
| C_16:0_ | 25.6 | 18.39 | 16.25 | 20.7 | 17.8 | 19.79 | 20.2 | 19.8 |
| C_17:0_ cyclo | 12.2 | 7.33 | ND | 6.0 | - | - | 2.9 | - |
| Summed Feature 3* | 16.7 | 10.47 | 22.22 | 15.5 | 23.5 | 24.22 | 22.2 | 22.4 |
| Summed Feature 8* | 19.6 | 36.18 | 43.98 | ND | 33.7 | 34.35 | 34.5 | 38.5 |

* Strains: 1, Strain 25A3E^T^; 2, *P. chengduensis* T1624^T^ (Tao et al., 2014); 3, *P. indoloxydans* JCM 14246^T^ (Behera et al., 2018); 4, *P. oleovorans* DSM 1045^T^(Saha et al., 2010) ; 5, *P. solani* Sm006^T^ (Sawada et al., 2023); 6, *P. tohonis* TUM 18999^T^ (Yamada et al., 2021); 7, *P. daroniae* P18A^T^ (Bueno-Gonzalez et al., 2019); 8, *P. flavescens* NBRC 103044^T^ (Bueno-Gonzalez et al., 2019). Summed feature 3 corresponds to C_16:1_*ω7c*/C_16:1_*ω6c*; summed feature 8 corresponds to C_18:1_ ω7c and/or C_18:1_ ω6c; -, trace amount (<0.5 %); ND, Not detected.

**Supplementary Table 2.** Genome characteristics of strain 25A3E^T^ and the closely related species.

| Characteristics | 1 | 2 | 3 | 4 | 5 | 6 | 7 | 8 |
| --- | --- | --- | --- | --- | --- | --- | --- | --- |
| Genome size (Mb) | 4.7 | 5.6 | 5.2 | 4.9 | 6.7 | 6.8 | 5.6 | 5.8 |
| G+C (%) | 65.5 | 62.5 | 62 | 62 | 66.5 | 66.5 | 62 | 63.5 |
| Genes (Total) | 4,415 | 5,194 | 5,134 | 4,743 | 6,050 | 6,222 | 5,142 | 5,407 |
| CDSs (Total) | 4,346 | 5,113 | 5,076 | 4,667 | 5,971 | 6,141 | 5,074 | 5,344 |
| Genes (coding) | 4,272 | 5,056 | 4,772 | 4,387 | 5,784 | 6,093 | 4,989 | 5,266 |
| rRNAs | 7 | 12 | 6 | 11 | 12 | 12 | 7 | 3 |
| tRNAs | 58 | 65 | 47 | 60 | 63 | 64 | 57 | 55 |
| ncRNAs | 4 | 4 | 5 | 5 | 4 | 5 | 4 | 5 |
| GenBank accession number | GCA_041027545.1 | GCA_023093875.1 | GCA_003052605.1 | GCA_002197815.1 | GCA_026072635.1 | GCA_012767755.2 | GCA_004327275.1 | GCA_002091575.1 |

**Supplementary Table 3.** The results of protein and pathway annotation generated by METABOLIC-G.

**Supplementary Table 4.** Genes involved in cold-stress response of strain 25A3E^T^.

**Supplementary Table 5.** Genes involved in organic matter degradation of strain 25A3E^T^.

**Supplementary Table 6.** Genes involved in nitrogen removal pathway of strain 25A3E^T^.

**References:**

Behera, P., Mahapatra, M., Seuylemezian, A., Vaishampayan, P., Ramana, V.V., Joseph, N., et al. (2018). Taxonomic description and draft genome of *Pseudomonas sediminis* sp. nov., isolated from the rhizospheric sediment of Phragmites karka. *Journal of Microbiology* 56**,** 458-466. doi: 10.1007/s12275-018-7549-x.

Bueno-Gonzalez, V., Brady, C., Denman, S., Plummer, S., Allainguillaume, J., and Arnold, D. (2019). *Pseudomonas daroniae* sp. nov. and *Pseudomonas dryadis* sp. nov., isolated from pedunculate oak affected by acute oak decline in the UK. *Int. J. Syst. Evol. Microbiol.* 69(11)**,** 3368-3376. doi: 10.1099/ijsem.0.003615.

Saha, R., Spröer, C., Beck, B., and Bagley, S. (2010). *Pseudomonas oleovorans* subsp. lubricantis subsp. nov., and reclassification of Pseudomonas pseudoalcaligenes ATCC 17440^T^ as later synonym of Pseudomonas oleovorans ATCC 8062^T^. *Curr. Microbiol.* 60**,** 294-300. doi: 10.1007/s00284-009-9540-6.

Sawada, H., Takeuchi, K., Someya, N., Morohoshi, T., and Satou, M. (2023). *Pseudomonas solani* sp. nov. isolated from the rhizosphere of eggplant in Japan. *Int. J. Syst. Evol. Microbiol.* 73(6)**,** 005942. doi: 10.1099/ijsem.0.005942.

Tao, Y., Zhou, Y., He, X., Hu, X., and Li, D. (2014). *Pseudomonas chengduensis* sp. nov., isolated from landfill leachate. *Int. J. Syst. Evol. Microbiol.* 64(Pt_1)**,** 95-100. doi: 10.1099/ijs.0.050294-0.

Yamada, K., Sasaki, M., Aoki, K., Nagasawa, T., Murakami, H., Ishii, M., et al. (2021). *Pseudomonas tohonis* sp. nov., isolated from the skin of a patient with burn wounds in Japan. *Int. J. Syst. Evol. Microbiol.* 71(11)**,** 005115. doi: 10.1099/ijsem.0.005115.
